# Mediator subunit MDT-15/MED15 and Nuclear Receptor HIZR-1/HNF4 cooperate to regulate toxic metal stress responses in *Caenorhabditis elegans*

**DOI:** 10.1371/journal.pgen.1008508

**Published:** 2019-12-09

**Authors:** Naomi Shomer, Alexandre Zacharie Kadhim, Jennifer Margaret Grants, Xuanjin Cheng, Deema Alhusari, Forum Bhanshali, Amy Fong-Yuk Poon, Michelle Ying Ya Lee, Anik Muhuri, Jung In Park, James Shih, Dongyeop Lee, Seung-Jae V. Lee, Francis Christopher Lynn, Stefan Taubert

**Affiliations:** 1 Graduate Program in Medical Genetics, The University of British Columbia, Vancouver, British Columbia, Canada; 2 Centre for Molecular Medicine and Therapeutics, The University of British Columbia, Vancouver, British Columbia, Canada; 3 British Columbia Children's Hospital Research Institute, Vancouver, British Columbia, Canada; 4 Department of Life Sciences, School of Interdisciplinary Bioscience and Bioengineering, Pohang University of Science and Technology, Pohang, Gyeongbuk, South Korea; 5 Department of Biological Sciences, Korea Advanced Institute of Science and Technology, Yuseong-Gu, Daejeon, South Korea; 6 Department of Surgery, The University of British Columbia, Vancouver, British Columbia, Canada; 7 Department of Medical Genetics, The University of British Columbia, Vancouver, British Columbia, Canada; Joslin Diabetes Center, UNITED STATES

## Abstract

Zinc is essential for cellular functions as it is a catalytic and structural component of many proteins. In contrast, cadmium is not required in biological systems and is toxic. Zinc and cadmium levels are closely monitored and regulated as their excess causes cell stress. To maintain homeostasis, organisms induce metal detoxification gene programs through stress responsive transcriptional regulatory complexes. In *Caenorhabditis elegans*, the MDT-15 subunit of the evolutionarily conserved Mediator transcriptional coregulator is required to induce genes upon exposure to excess zinc and cadmium. However, the regulatory partners of MDT-15 in this response, its role in cellular and physiological stress adaptation, and the putative role for mammalian MED15 in the metal stress responses remain unknown. Here, we show that MDT-15 interacts physically and functionally with the Nuclear Hormone Receptor HIZR-1 to promote molecular, cellular, and organismal adaptation to cadmium and excess zinc. Using gain- and loss-of-function mutants and qRT-PCR and reporter analysis, we find that *mdt-15* and *hizr-1* cooperate to induce zinc and cadmium responsive genes. Moreover, the two proteins interact physically in yeast-two-hybrid assays and this interaction is enhanced by the addition of zinc or cadmium, the former a known ligand of HIZR-1. Functionally, *mdt-15* and *hizr-1* mutants show defective storage of excess zinc in the gut and are hypersensitive to zinc-induced reductions in egg-laying. Furthermore, *mdt-15* but not *hizr-1* mutants are hypersensitive to cadmium-induced reductions in egg-laying, suggesting potential divergence of regulatory pathways. Lastly, mammalian MDT-15 orthologs bind genomic regulatory regions of metallothionein and zinc transporter genes in a cadmium and zinc-stimulated fashion, and human MED15 is required to induce a metallothionein gene in lung adenocarcinoma cells exposed to cadmium. Collectively, our data show that *mdt-15* and *hizr-1* cooperate to regulate cadmium detoxification and zinc storage and that this mechanism is at least partially conserved in mammals.

## Author summary

In all life forms, zinc plays essential cellular and molecular roles. However, as with all molecules, excess zinc is hazardous, as is the presence of the physically and chemically highly similar cadmium. Organisms have evolved mechanisms to protect themselves against cadmium and high levels of zinc. Using the roundworm *Caenorhabditis elegans*, we previously showed that the Mediator subunit MDT-15 is essential in many cytoprotective responses including that against excess zinc or cadmium. Here, we find that the nuclear receptor HIZR-1, shown previously to convey high zinc detoxification, works together with MDT-15 to protect worms against high zinc as well as cadmium by cooperatively activating a cytoprotective gene expression program. Notably, the two proteins bind physically in a fashion that is enhanced by exogenous zinc or cadmium; these metals thus appear to act like stereotypical hormones by promoting a nuclear receptor–coregulator interaction. Exploring the evolutionary conservation of this mechanism, we find that MDT-15’s orthologues bind and regulate zinc and cadmium responsive genes in mammalian cell lines. This provides the first evidence that mammalian MED15 functions in stress response pathways, establishing it as a cytoprotective regulator across eukaryotes.

## Introduction

In their habitats, biological organisms encounter many metals, including essential micronutrients such as zinc, iron, copper, and manganese, and toxic metals such as cadmium, mercury, lead, and arsenic. Zinc is an essential trace element that plays a crucial role in numerous cellular and physiological processes [1]. It has a structural role in metabolic enzymes, growth factors, and zinc finger proteins, and is also an enzymatic cofactor and a signaling molecule [2,3]. Accordingly, zinc is necessary for the function of approximately 10% of proteins in the human proteome and approximately 8% of proteins in the nematode worm *Caenorhabditis elegans* [4]. In line with its requirement in diverse proteins, zinc deficiency causes a broad range of symptoms and dysfunctions in humans, such as skin and eye lesions, thymic atrophy, diarrhea, defective wound healing, and others [5,6]. *Vice versa*, although rare and generally associated with decreased copper uptake and associated deficiency, exposure to high doses of zinc is also detrimental, as it has toxic effects, causes cell stress, and alters physiological programs such as systemic growth, immune responses, and neuro-sensory and endocrine functions [5].

Unlike zinc, cadmium is a non-essential toxic metal encountered by biological organisms as a naturally occurring and industrial environmental contaminant. Cadmium has no known function in biological systems, and exposure causes intracellular damage along with the production of reactive oxygen species [7]. In humans, cadmium exposure can result in respiratory disease, kidney damage, neurological disorders, and various types of cancers [7,8]. Interestingly, zinc and cadmium share a similar electron configuration. Cadmium may therefore substitute for zinc at the molecular level, for example as an enzyme cofactor, consequently reducing or abrogating normal protein function [9].

Another consequence of the elemental similarity is that the biological responses to and the systemic detoxification of zinc and cadmium are similar [2,5,10,11]. Key detoxification and homeostasis components include metal-sequestering proteins such as metallothioneins (MTs), which bind a wide range of metals including cadmium, lead, zinc, copper, and others [12]. Other important detoxification and homeostasis components include the cation diffusion facilitators (CDFs; also known as zinc transporter (ZnT) or solute carrier 30 (SLC30) family proteins) that transport zinc into the cytoplasm, and the Zrt- and Irt-like proteins (ZIP; aka SLC39A family proteins) that transport zinc out of the cytoplasm [13,14].

To maintain homeostasis in the face of changing metal levels, transcriptional regulatory mechanisms adjust gene expression as needed. Metal-responsive transcription factor-1 (MTF-1) is a transcription factor that is evolutionarily conserved from insects to humans. It binds metal responsive elements (MREs) in the promoters of target genes (e.g. metallothioneins) and regulates their expression when certain metals are in excess [15–17]. MTF-1 directly senses zinc with its six zinc fingers and with an acidic, zinc-responsive transcriptional activation domain; related metals such as cadmium appear not to directly bind MTF-1 and instead are likely detected indirectly through the altered availability of zinc. However, additional transcription factors and/or activation mechanism must control gene expression in response to excess metal, as some genes are regulated independently of MTF-1 and MREs in these conditions [16,18]. Indeed, the *C*. *elegans* genome features MREs but lacks a detectable MTF-1 ortholog. In this worm, the activation of genes by high zinc levels instead requires the High Zinc Activated (HZA) element and the HZA-binding Nuclear Hormone Receptor high-zinc–activated nuclear receptor 1 (HIZR-1; aka NHR-33) [19,20]. However, whether HIZR-1’s role extends beyond zinc detoxification is not known. HIZR-1 is a sequence homolog of mammalian Hepatocyte Nuclear Factor 4 (HNF4) [20,21], but whether HNF4 proteins regulate gene expression in response to high metal concentrations in mammals remains unknown.

To effectively and specifically activate gene expression, transcription factors require accessory proteins termed coregulators [22–24]. One important coregulator is Mediator, a ~30 protein subunit complex that is conserved from yeast to human [25,26]. Individual Mediator subunits selectively engage transcription factors and thus regulate specific developmental and physiological gene programs. In particular, Mediator subunit MDT-15/Med15 and Mediator kinase cyclin dependent kinase 8 (CDK-8) are required for many stress and adaptive responses across species [26–31]. In the context of metal responsive transcription, *Drosophila melanogaster* MTF-1 requires Mediator for gene activation via MREs [17,32]. In *C*. *elegans*, we showed that Mediator subunit *mdt-15* is required for the induction of zinc and cadmium responsive genes [33]. Others found that zinc-dependent activation of three extrachromosomal promoter reporters required *mdt-15*; these reporters contain the HIZR-1 binding HZA element as well as a GATA element that is bound by GATA factors and is a general feature of zinc-responsive promoters [19]. As MDT-15 is a known coregulator of HNF4-like NHRs in *C*. *elegans* [34–37], this suggests that MDT-15 may cooperate with the HZA-binding HIZR-1 to adapt gene expression in response to high zinc in *C*. *elegans*.

Here, we tested whether MDT-15 and HIZR-1 cooperate to regulate zinc and cadmium responsive transcription in *C*. *elegans* and whether MED15, the mammalian ortholog of MDT-15, also participates in zinc and cadmium responses. We were also interested in studying the role of the Mediator kinase CDK-8 in this context, because: (i) it also regulates stress responses [30,31]; (ii) CDK-8 antagonizes MDT-15 in *C*. *elegans* [38]; and (iii) Cdk8 antagonizes Med15 in yeast, including in the low iron adaptive response [39]. Thus, we also assessed the function of *C*. *elegans cdk-8* in zinc and cadmium responsive transcription. Using genetic, molecular, cytological, and functional assays, we find that MDT-15 and HIZR-1 interact physically and functionally in zinc and cadmium stress responses, and that mammalian MED15 is recruited to zinc and cadmium responsive genes and required for cadmium-induced gene expression.

## Results

### *mdt-15* and *cdk-8* are necessary for cadmium- and zinc-induced gene activation

**Fig 1:**
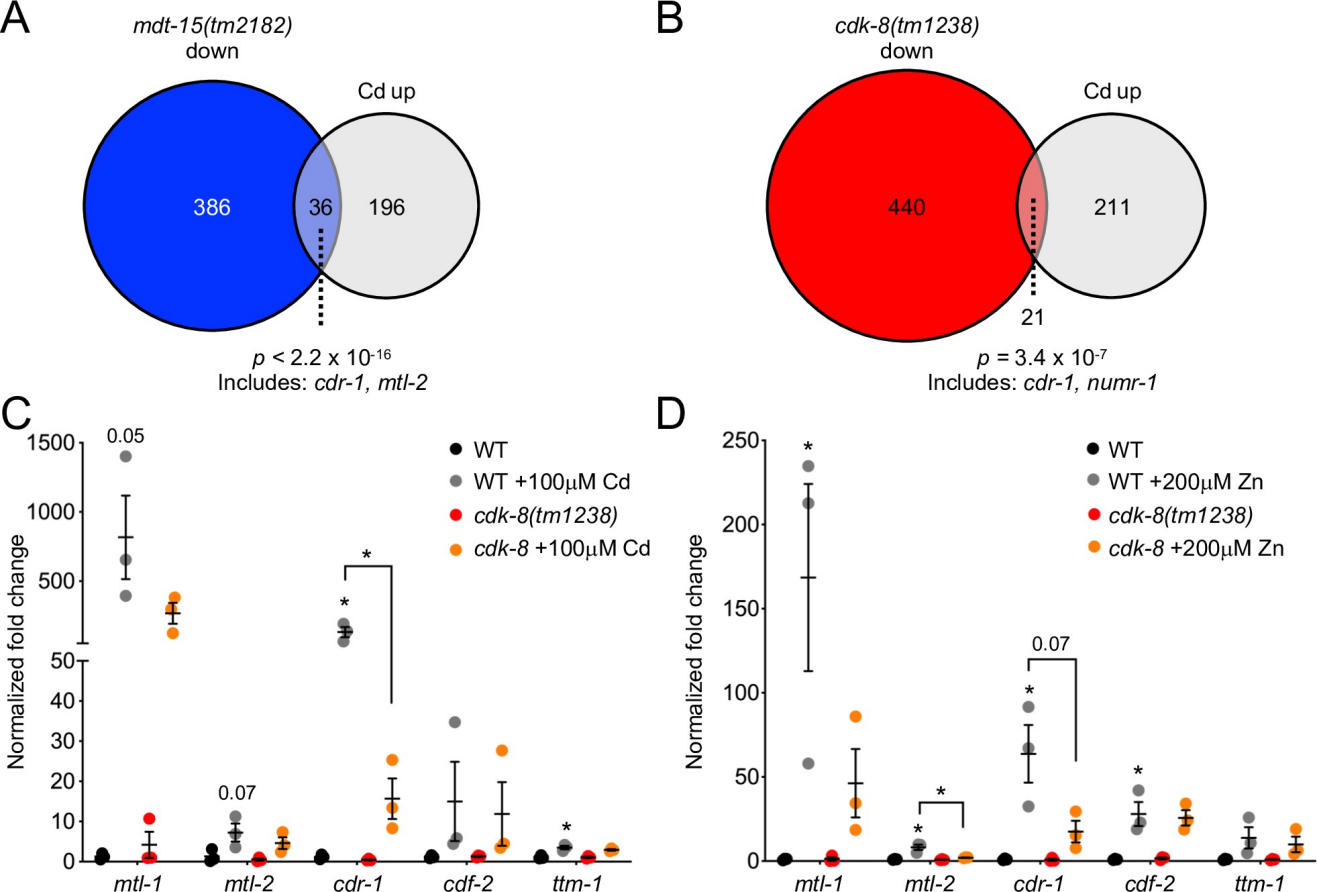
Mediator subunits *mdt-15* and *cdk-8* are required for cadmium and zinc stress responses. **[A]** The Venn diagram depicts the overlap between genes downregulated in the *mdt-15(tm2182)* mutant and genes induced by cadmium (identified in [43]). Statistical significance was assessed by Fisher’s exact test. Shared genes include *cdr-1* and *mtl-2*, which are known to function in cadmium biology [40,41]. See S1 Table for details. **[B]** The Venn diagram depicts the overlap between genes downregulated in the *cdk-8(tm1238)* mutant and genes induced by cadmium. Statistical significance was assessed by Fisher’s exact test. Shared genes include *cdr-1* and *numr-1*, which are known to function in cadmium biology [40,42,44]. See S1 Table for details. **[C-D]** qRT-PCR analysis of zinc and cadmium-responsive genes in wild-type (WT) and *cdk-8(tm1238)* mutant worms grown on 100μM cadmium for 4 hours [C] or on 200μM zinc for 16 hours [D]. Graphs show fold induction, normalized to the average of unsupplemented WT mRNA levels. Error bars: SEM (n *=* 3 independent repeats). Statistical analysis: * *p <* 0.05, unpaired t-test comparing WT supplemented worms to WT supplemented worms, except for comparisons indicated by lines (note, only significant or near-significant (p < 0.075) values are shown).

We previously showed that *mdt-15* is required to induce the mRNA levels of several genes in response to high levels of zinc and cadmium [33]. In line with this finding, genes downregulated in *mdt-15(tm2182)* hypomorph mutants showed significant overlap with cadmium-induced genes (Fig 1A and S1 Table). Similarly, cadmium-induced genes overlap significantly with genes downregulated in *cdk-8 (tm1238)* null mutants (Fig 1B and S1 Table), suggesting that *cdk-8* might play a similar role as *mdt-15*. Cadmium-inducible genes deregulated in the Mediator subunit mutants include *cdr-1*, *mtl-1*, and *numr-1*, which are necessary for resistance against cadmium toxicity in *C*. *elegans* [40–42]. Notably, *cdr-1* was downregulated in both mutants (S1 Table).

To test the requirement of *cdk-8* in cadmium-induced detoxification and to assess its role in the zinc response, we used quantitative reverse transcription PCR (qRT-PCR) to measure the mRNA levels of metallothioneins (*mtl-1*, *mtl-2*), zinc transporters (ZnT) implicated in cadmium and zinc detoxification (*ttm-1*, *cdf-2*), and the cadmium responsive gene *cdr-1* [10,20,33]. We studied these genes because their mRNA levels respond to changing cadmium and/or zinc levels and are regulated by HIZR-1 and/or MDT-15 [19,20,33,40]; we were especially interested in *cdr-1* because it was identified by our analysis in Fig 1. Comparing wild-type worms and *cdk-8(tm1238)* mutants, we observed that loss of *cdk-8* significantly reduced the induction of *cdr-1* mRNA by cadmium and of *mtl-2* mRNA by zinc (Fig 1C and 1D). Thus, *cdk-8* is required for maximal gene expression in response to zinc or cadmium, but this requirement is less prevalent and substantial than the one we previously observed for *mdt-15* [33].

### *hizr-1* and *elt-2* are required to induce the *cdr-1* promoter

**Fig 2:**
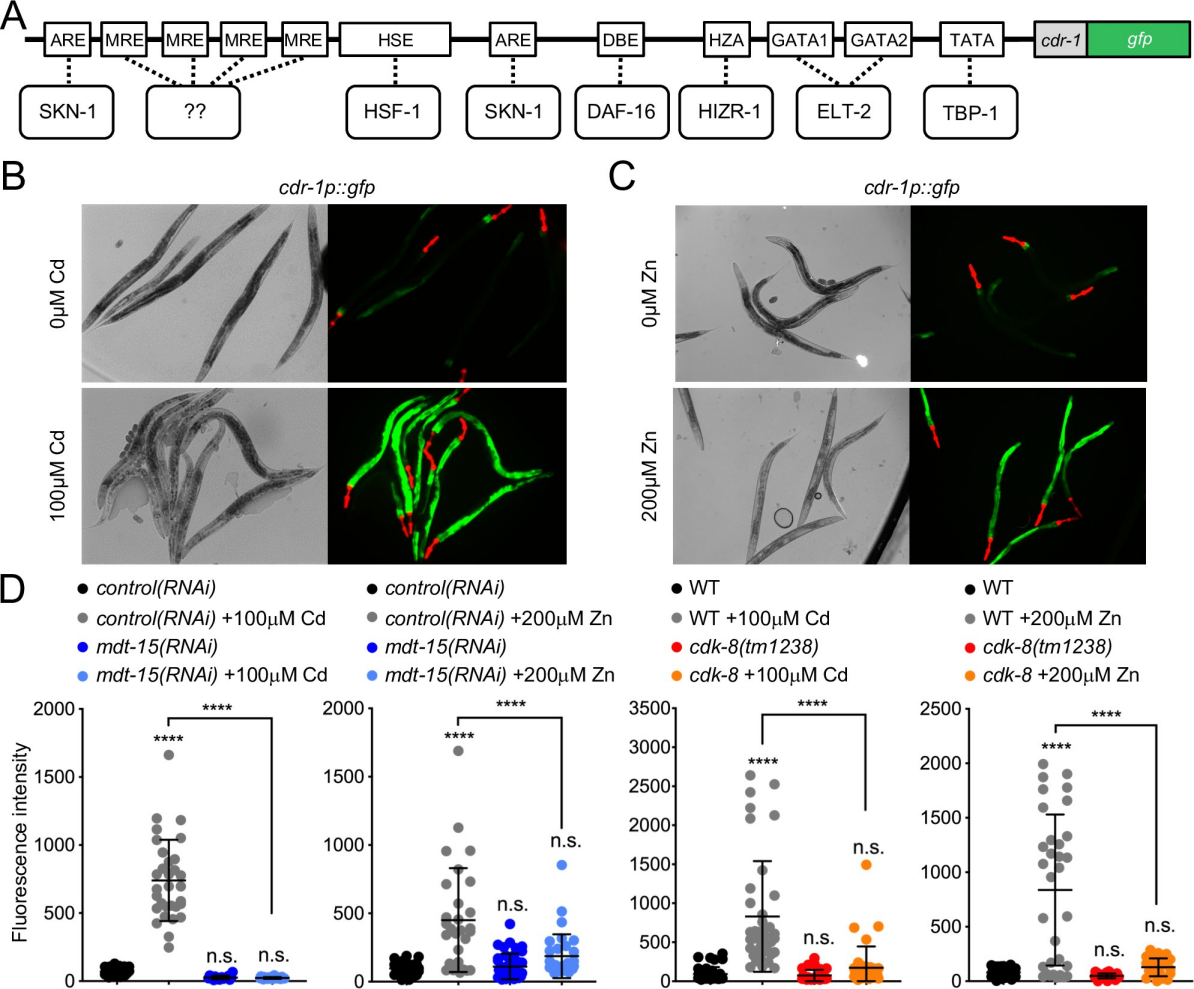
The *cdr-1p*::*gfp* transcriptional reporter is induced by 100μM cadmium and 200μM zinc in *cdk-8–* and *mdt-15*–dependent fashion. **[A]** Illustration of the *cdr-1p*::*gfp* reporter and putative regulatory DNA elements in the *cdr-1* promoter; note, the diagram is not to scale. For details, see text. **[B, C]** Representative micrographs of worms bearing the *cdr-1p*::*gfp* and *myo-2p*::*mCherry* (transgenic marker) transcriptional reporters, without and with exposure to [A] 100μM cadmium and [B] 200μM zinc. **[D]** The graphs show the average fluorescence intensity (arbitrary units, A.U.) of worms bearing the *cdr-1p*::*gfp* transcriptional reporter. The two panels on the left show WT worms bearing the *cdr-1p*::*gfp* transcriptional reporter fed either control or *mdt-15* RNAi and exposed for four hours to 0μM or 100μM Cd or 0μM or 200μM Zn (control RNAi 0μM Cd: n = 32 worms total from 3 independent repeats; control RNAi 100μM Cd: n = 31 worms total from 3 independent repeats: *mdt-15* RNAi 0μM Cd: n = 19 worms total from 3 independent repeats; *mdt-15* RNAi 100μM Cd: n = 32 worms total from 3 independent repeats; control RNAi 0μM Zn: n = 30 worms 3 total from independent repeats; control RNAi 200μM Zn; n = 29 worms total from 3 independent repeats; *mdt-15* RNAi 0μM Zn: n = 42 worms total from 3 independent repeats: *mdt-15* RNAi 200μM Zn: n = 33 worms total from 3 independent repeats). The two panels on the right show WT and *cdk-8(tm1238)* worms exposed for four hours to 0μM or 100μM cadmium (Cd) or 0μM or 200μM zinc (Zn) (WT 0μM Cd: n = 39 worms total from 3 independent repeats; WT 100μM Cd: n = 39 worms total from 3 independent repeats; *cdk-8(tm1238)* 0μM Cd: n = 29 worms total from 3 independent repeats; *cdk-8(tm1238)* 100μM Cd: n = 38 worms total from 3 independent repeats; WT 0μM Zn: n = 31 worms total from 3 independent repeats; WT 200μM Zn: n = 32 worms total from 3 independent repeats; *cdk-8(tm1238)* 0μM Zn: n = 31 worms total from 3 independent repeats; *cdk-8(tm1238)* 200μM zinc: n = 24 worms total from 3 independent repeats). Error bars: standard deviation. Statistical analysis: **** *p* < 0.0001, n.s. = not significant, two-way ANOVA, multiple comparisons, Tukey correction; comparisons are to WT or *control(RNAi)* unless indicated by lines.

To delineate the mechanism of MDT-15 and CDK-8 driven, cadmium and zinc responsive transcription, we generated a transcriptional *cdr-1p*::*gfp* reporter, encompassing 2.8 kb of the putative *cdr-1* promoter (Fig 2A). We chose *cdr-1* as a model because it is highly cadmium and zinc responsive and requires both *mdt-15* and *cdk-8* for activation [10,33,40] (Fig 1 and S1 Table), suggesting that it might be a good tool to identify DNA regulatory elements and cognate transcription factors that cooperate with these Mediator subunits. As expected [10,33,40], we observed weak basal expression of this reporter, but substantial induction of fluorescence by 200μM zinc and 100μM cadmium (Fig 2B and 2C). Expression was primarily localized to the intestine (Fig 2B and 2C), as expected [10,40]. Knockdown of *mdt-15* by feeding RNA interference (RNAi) caused a significant decrease of *cdr-1p*::*gfp* fluorescence induction by cadmium and zinc (Fig 2D). Similarly, *cdk-8(tm1238); cdr-1p*::*gfp* worms showed a significant decrease in cadmium and zinc-induced fluorescence (Fig 2D).

**Fig 3:**
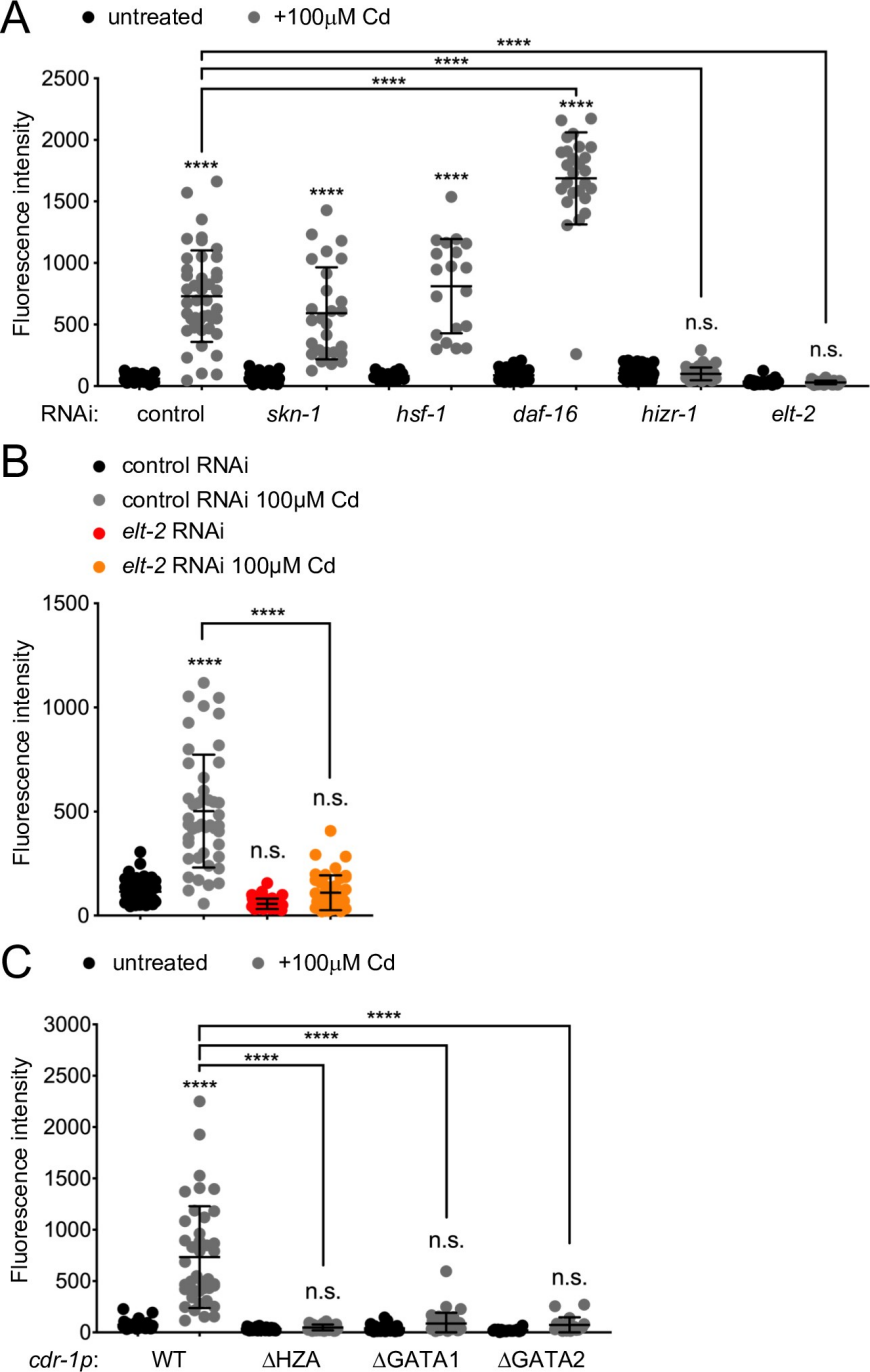
The TFs *hizr-1* and *elt-2* and their cognate promoter elements are required for the cadmium stress responses. **[A]** The graph shows the average fluorescence intensity (arbitrary units, A.U.) of worms bearing the *cdr-1p*::*gfp* transcriptional reporter before and after a 0μM or 100μM cadmium (Cd) exposure (four hours), and fed either control or transcription factor RNAi, as indicated (control RNAi 0μM Cd: n = 51 worms total from 4 independent repeats; control RNAi 100μM Cd: n = 43 worms total from 4 independent repeats; *skn-1* RNAi 0μM Cd: n = 39 worms total from 3 independent repeats; *skn-1* RNAi 100μM Cd: n = 27 worms total from 3 independent repeats; *hsf-1* RNAi 0μM Cd: n = 22 worms total from 2 independent repeats; *hsf-1* RNAi 100μM Cd: n = 19 worms total from 2 independent repeats; *daf-16* RNAi 0μM Cd: n = 27 worms total from 2 independent repeats; *daf-16* RNAi 100μM Cd: n = 26 worms total from 2 independent repeats; *hizr-1* RNAi 0μM Cd: n = 50 worms total from 3 independent repeats; *hizr-1* RNAi 100μM Cd: n = 40 worms total from 3 independent repeats; *elt-2* RNAi 0μM Cd: n = 22 worms total from 3 independent repeats; *elt-2* RNAi 100μM Cd: n = 37 worms total from 3 independent repeats). Error bars: standard deviation. Statistical analysis: **** *p* < 0.0001, n.s. = not significant, two-way ANOVA, multiple comparisons, Tukey correction; comparisons are to *control(RNAi)* 0μM Cd unless indicated by lines. **[B]** The graph shows the average fluorescence intensity (arbitrary units, A.U.) of day-3 old adult worms bearing the *cdr-1p*::*gfp* transcriptional reporter, fed either control or *elt-2* RNAi during the first two days of adulthood and then exposed to 0μM or 100μM cadmium (Cd) for four hours (control RNAi 0μM Cd: n = 48 worms total from 3 independent repeats; control RNAi 100μM Cd: n = 45 worms total from 3 independent repeats; *elt-2* RNAi 0μM Cd: n = 48 worms total from 3 independent repeats; *elt-2* RNAi on 100μMCd: n = 43 worms total from 3 independent repeats). Error bars: standard deviation. Statistical analysis: **** *p* < 0.0001, n.s. = not significant, two-way ANOVA, multiple comparisons, Tukey correction; comparisons are to control RNAi 0μM Cd unless indicated by lines. **[C]** The graph shows the average fluorescence intensity (arbitrary units, A.U.) of worms bearing the following *cdr-1p*::*gfp* reporter variants: wild-type, WT; mutated HZA element, *cdr-1pΔHZA*; mutated GATA elements, *cdr-1pΔGATA1* or *cdr-1pΔGATA2*. All mutants were assessed after a four-hour 0μM or 100μM cadmium (Cd) exposure (WT 0μM Cd: n = 37 worms total from 4 independent repeats; WT 100μM Cd: n = 41 worms total from 4 independent repeats; *cdr-1pΔHZA* 0μM Cd: n = 42 worms total from 2 independent repeats; *cdr-1PΔHZA* 100μM Cd: n = 30 worms total from 2 independent repeats; *cdr-1pΔGATA1* 0μM Cd: n = 32 worms total from 3 independent repeats; *cdr-1pΔGATA1* 100μM Cd: n = 37 worms total from 3 independent repeats; *cdr-1pΔGATA2* 0μM Cd: n = 19 worms total from 2 independent repeats; *cdr-1PΔGATA2* 100μM Cd: = 19 worms total from 2 independent repeats). Error bars: standard deviation. Statistical analysis: **** *p* < 0.0001, n.s. = not significant, two-way ANOVA, multiple comparisons, Tukey correction; comparisons are to WT unless indicated by lines.

To identify transcription factors that cooperate with *cdk-8* and *mdt-15* to regulate cadmium and zinc responsive transcription, we searched for DNA regulatory elements in the *cdr-1* promoter. We identified candidate elements recognized by SKN-1/Nrf2 (antioxidant response element, ARE), HSF-1 (heat shock response element, HSE), DAF-16/FOXO (DAF-16 binding element, DBE), ELT-2 (GATA element), and HIZR-1 (HZA element), as well as four MREs, which no *C*. *elegans* transcription factor is yet known to bind (Fig 2A). RNAi analysis revealed that *skn-1*, *hsf-1*, and *daf-16* are not required for *cdr-1* induction by cadmium; *daf-16* depletion actually hyper-induced the *cdr-1p*::*gfp* promoter (Fig 3A). In contrast, knocking down *elt-2* or *hizr-1* abrogated fluorescence induction (Fig 3A). As *elt-2* is required for intestinal development [45], we examined post-developmental *elt-2* knockdown, which also caused abrogation of basal and cadmium-induced *cdr-1p*::*gfp* expression (Fig 3B). Thus, *elt-2* is required at the *cdr-1* promoter independently of its role in intestine development.

We confirmed the requirements for *elt-2* and *hizr-1* by site-directed mutagenesis of their cognate DNA elements. We generated substitution mutations in the HZA (*ΔHZA*) or GATA sites (*ΔGATA1* and *ΔGATA2*) of the *cdr-1p*::*gfp* reporter (Fig 3C). Each mutation individually caused a significant decrease in cadmium-induced promoter activity compared to the wild-type *cdr-1p*::*gfp* reporter (Fig 3C). Collectively, these data show that Mediator subunits MDT-15 and CDK-8 and the transcription factors ELT-2 and HIZR-1 are required to control expression from the cadmium/zinc-inducible *cdr-1* promoter.

### *mdt-15* and *hizr-1* function is co-dependent

The above data suggest that MDT-15 and/or CDK-8 might interact functionally and physically with HIZR-1 and/or ELT-2 to activate metal-induced transcription. However, *cdk-8* is required only for *cdr-1* induction by cadmium and zinc and for *mtl-2* induction by zinc (Figs 1 and 2); in contrast, *mdt-15* is required to induce *mtl-1*, *mtl-2*, *cdr-1*, and *cdf-2* in response to both metals (Fig 2 and [33]). Thus, the role of CDK-8 appears weaker compared MDT-15, and we focused hereafter on MDT-15 and its putative interaction with HIZR-1.

**Fig 4:**
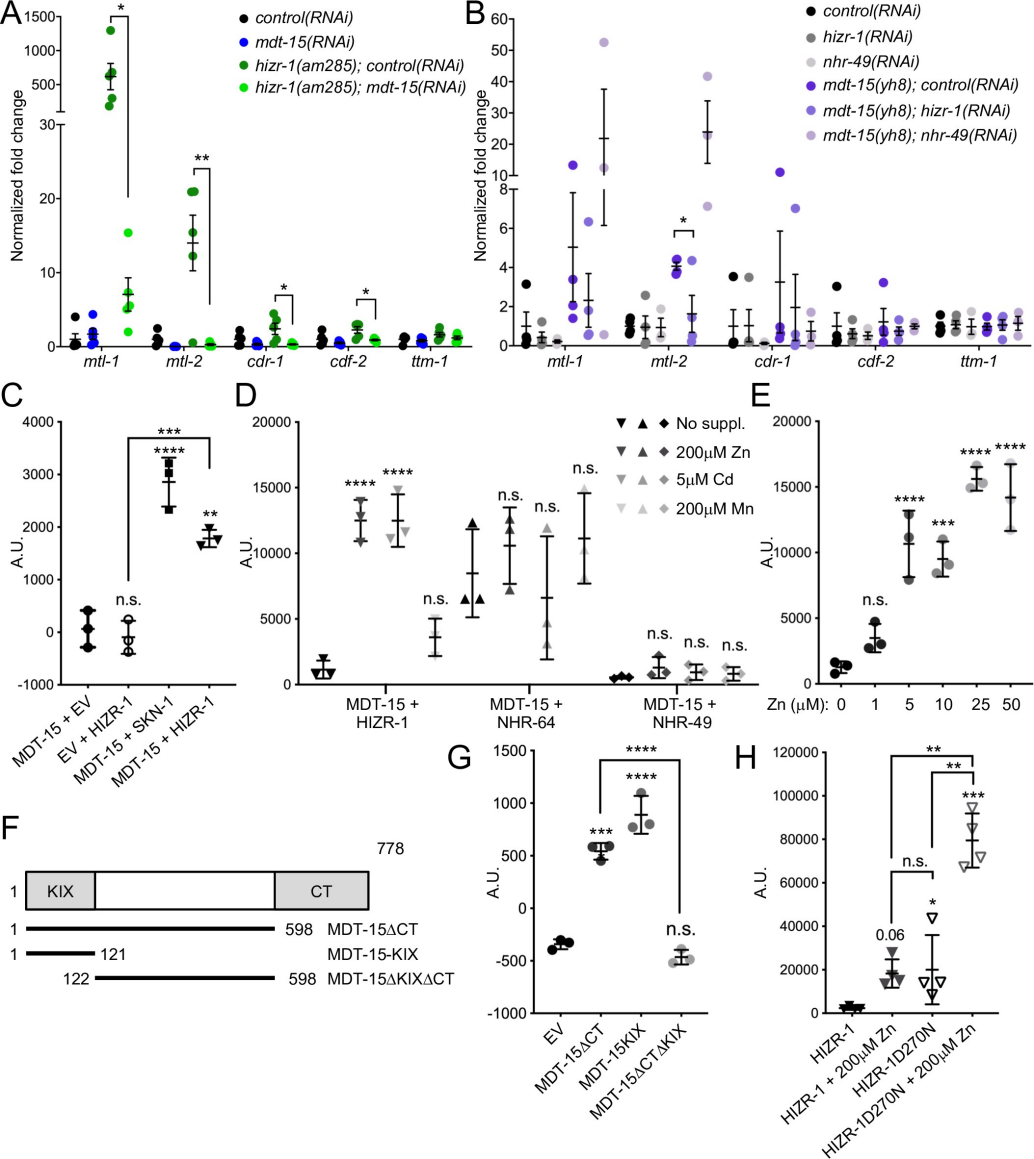
MDT-15 and HIZR-1 are co-dependent for metal responsive gene induction and physically bind in cadmium and zinc-enhanced fashion. **[A-B]** qRT-PCR analysis of zinc and cadmium-responsive genes. The graphs show fold-inductions of mRNAs normalized to the average of wild-type *control(RNAi)* worms. Error bars: SEM. Statistical analysis: * *p <* 0.05, ** *p <* 0.01, unpaired Student’s t-test for indicated comparisons. [A] Comparison of wild-type and *hizr-1(am285)* gf worms grown on control or *mdt-15* RNAi (n *=* 5 independent repeats). [B] Comparison of wild-type and *mdt-15(yh8)* gf worms grown on control, *hizr-1*, or *nhr-49* RNAi (n = 3–4 independent repeats). **[C-E]** Protein-protein interaction analysis using the Y2H system. Graphs show average interaction strength (arbitrary units, A.U.). Error bars: standard deviation. [C] Interaction between MDT-15 and HIZR-1, with Empty Vector (EV)–HIZR-1 and MDT-15–EV as negative controls, and MDT-15–SKN-1c as a positive control (n = 3 independent repeats). Statistical analysis: n.s. = not significant; ** p < 0.01, *** p < 0.001, **** p < 0.0001, One-way ANOVA, multiple comparisons, Tukey correction. All comparisons for “MDT-15 + EV” unless indicated by lines. Expression of HIZR-1 prey fusion proteins is shown in panel A of S1 Fig. [D] Average interaction strength between MDT-15 and HIZR-1; MDT-15 and NHR-64; and MDT-15 and NHR-49, all with no treatment, or treated with 200μM zinc, 5μM cadmium, or 200μM manganese (n = 3 independent repeats). Statistical analysis: n.s. = not significant; **** p < 0.0001, One-way ANOVA, multiple comparisons, Sidak correction; all comparisons to the pertinent “no supplement” control. Expression of HIZR-1, NHR-64, and NHR-49 prey fusion proteins is shown in panel B of S1 Fig. [E] Interaction between MDT-15 bait and HIZR-1 prey without zinc supplementation and with 1, 5, 10, 25, or 50μM supplementary zinc (n = 3 independent repeats). Statistical analysis: n.s. = not significant; *** p < 0.001, **** p < 0.0001, One-way ANOVA, multiple comparisons, Dunnett correction; all comparisons to “0μM zinc”. Expression of HIZR-1 prey fusion proteins is shown in panel C of S1 Fig. **[F]** The diagram shows the MDT-15 deletions analyzed for HIZR-1 binding in [G]. Numbers indicate amino acids; the N-terminal, NHR-binding KIX-domain (“KIX”) and the C-terminal autoactivation domain (“CT”) are shaded gray. **[G-H]** Protein-protein interaction analysis using the Y2H system. Graphs show average interaction strength (arbitrary units, A.U.). Error bars: standard deviation. [G] Interaction between empty vector (EV) or MDT-15 mutant (MDT-15ΔCT, MDT-15-KIX, or MDT-15ΔKIXΔCT) baits and a full-length WT HIZR-1 prey (n = 3 independent repeats). Statistical analysis: n.s. = not significant, **** p < 0.0001, One-way ANOVA, multiple comparisons, Sidak correction; comparisons are to “MDT-15ΔCT” unless indicated by lines. Expression of HIZR-1 prey fusion proteins is shown in panel D of S1 Fig. [H] Interaction between HIZR-1 and MDT-15 or HIZR-1-D270N and MDT-15 with and without zinc treatment (n = 4 independent repeats). Statistical analysis: n.s. = not significant, ** p < 0.01, *** p < 0.001, One-way ANOVA, multiple comparisons, Sidak correction; comparisons to “HIZR-1” unless indicated by lines. Expression of HIZR-1 prey fusion proteins is shown in panel E of S1 Fig.

To examine the putative functional relationship between MDT-15 and HIZR-1, we studied the *hizr-1(am285)* D270N gain-of-function (gf) mutant that induces zinc responsive genes even in the absence of zinc [20]. In line with published data [20], qRT-PCR analysis revealed induction of *cdr-1*, *mtl-1*, *mtl-2*, and *cdf-2* in *hizr-1(am285)* mutants grown on control RNAi; importantly, *mdt-15* RNAi significantly reduced or abrogated these inductions (Fig 4A). Next, we studied the *mdt-15(et14)* P117L gf mutation that induces MDT-15 regulated lipid metabolism genes [46]. Because this mutation is closely linked to a *paqr-1(3410)* loss-of-function mutation [46], we used the *mdt-15(yh8)* strain, which was generated by CRISPR and carries P117L alone [47]. The *mdt-15(yh8)* gf mutation was sufficient to induce *mtl-2*, and RNAi experiments revealed that *mtl-2* induction required *hizr-1* (Fig 4B). In contrast, *nhr-49*, which cooperates with MDT-15 to activate lipid metabolism and stress response genes [34,36], was not required to induce *mtl-2* in the *mdt-15(yh8)* mutant (Fig 4B). We conclude that MDT-15 and HIZR-1 specifically cooperate to induce zinc and cadmium responsive genes.

### MDT-15 physically interacts with HIZR-1 in zinc-enhanced fashion in yeast two hybrid assays

The above data suggest that MDT-15 might interact physically with HIZR-1, as shown for other *C*. *elegans* HNF4-like NHRs [34,35]. To test whether HIZR-1 binds MDT-15, we used the yeast-two-hybrid (Y2H) system. First, we examined the interaction between a full length HIZR-1 prey and a MDT-15-ΔCT bait (aa 1–600); full-length MDT-15 (aa 1–780) autoactivates and cannot be used as bait in Y2H assays (see [48] for details). In these assays, MDT-15 and HIZR-1 showed a statistically significant interaction that was similar in strength to the interaction of the MDT-15-ΔCT bait with a SKN-1c prey (positive control [48]; Fig 4C; for expression of Y2H prey fusion proteins, see S1 Fig).

The HIZR-1 ligand binding domain (LBD) binds zinc in the micromolar range, suggesting that zinc is a *bona fide* ligand for HIZR-1 [20]. We hypothesized that the addition of zinc might enhance the interaction of HIZR-1 with MDT-15, as shown for other ligand-stimulated NHR-coregulator interactions. Indeed, in our Y2H assays, addition of zinc enhanced the interaction between MDT-15 and HIZR-1 (Fig 4D), with significant effects at low micromolar concentrations (Fig 4E). As *mdt-15* also contributes to cadmium-induced gene expression, we tested whether this metal also enhanced MDT-15 binding to HIZR-1 and found that this was the case (Fig 4D). In contrast, manganese did not enhance the binding of MDT-15 to HIZR-1 (Fig 4D).

NHRs contain a zinc-finger DNA binding domain (DBD), suggesting that zinc-stimulated binding to a coregulator such as MDT-15 might be a common feature of NHRs. To examine the specificity of zinc- and cadmium-stimulated MDT-15–NHR interaction, we used the Y2H system to examine whether these metals alter binding to two other known MDT-15 binding partners, NHR-64 and NHR-49 [34]. We found that NHR-64 interacts with MDT-15 as strongly as HIZR-1 does in the presence of zinc or cadmium, while NHR-49 binding to MDT-15 mimics the interaction of HIZR-1 and MDT-15 in the absence of zinc; importantly, neither NHR-64 nor NHR-49 binding to MDT-15 was altered by supplementation with zinc, cadmium, or manganese at the concentrations that significantly enhanced binding of HIZR-1 to MDT-15 (Fig 4D). We conclude that metal-stimulation of MDT-15 interaction is not a general feature of NHRs but specific to HIZR-1.

### Binding determinants in MDT-15 and HIZR-1

MDT-15 contains an N-terminal KIX-domain that binds several NHRs and the lipogenic transcription factor SBP-1 [34,35,49]. Thus, we hypothesized that MDT-15 might physically bind HIZR-1 through the KIX-domain (aa 1–124). To test this hypothesis, we used the Y2H system to assay binding of HIZR-1 to an MDT-15-KIX-domain and to an MDT-15ΔCT variant lacking the KIX-domain (MDT-15ΔKIXΔCT; aa 125–600; Fig 4F). The binding of HIZR-1 to the MDT-15-KIX-domain was similar in strength to the binding to MDT-15ΔCT; in contrast, MDT-15ΔKIXΔCT was unable to bind HIZR-1 (Fig 4G). This indicates that the KIX-domain is necessary and sufficient for the HIZR-1–MDT-15 interaction.

The *hizr-1(am285)* gf mutation is an aspartate 270 to asparagine (D270N) substitution that results in increased nuclear localization and constitutive activation of zinc responsive genes even in the absence of zinc [20]. Sequence comparison revealed that D270 is conserved in the HIZR-1 orthologs of four other species in the *Caenorhabditis* genus and in *Pristionchus pacificus* (S2 Fig), suggesting that it may be functionally important. We hypothesized that the D270N mutation might affect MDT-15 binding. To test this, we performed Y2H assays with a HIZR-1-D270N prey generated by site-directed mutagenesis. HIZR-1-D270N interacted more strongly with MDT-15 than did HIZR-1-WT, resembling in strength the WT HIZR-1–MDT-15 interaction in the presence of zinc (Fig 4H). Nevertheless, supplemental zinc further enhanced this interaction (Fig 4H), suggesting that, D270 does not mimic the effects of zinc-stimulated binding.

### *mdt-15(tm2182)* mutants display zinc storage defects and are hypersensitive to zinc and cadmium

**Fig 5:**
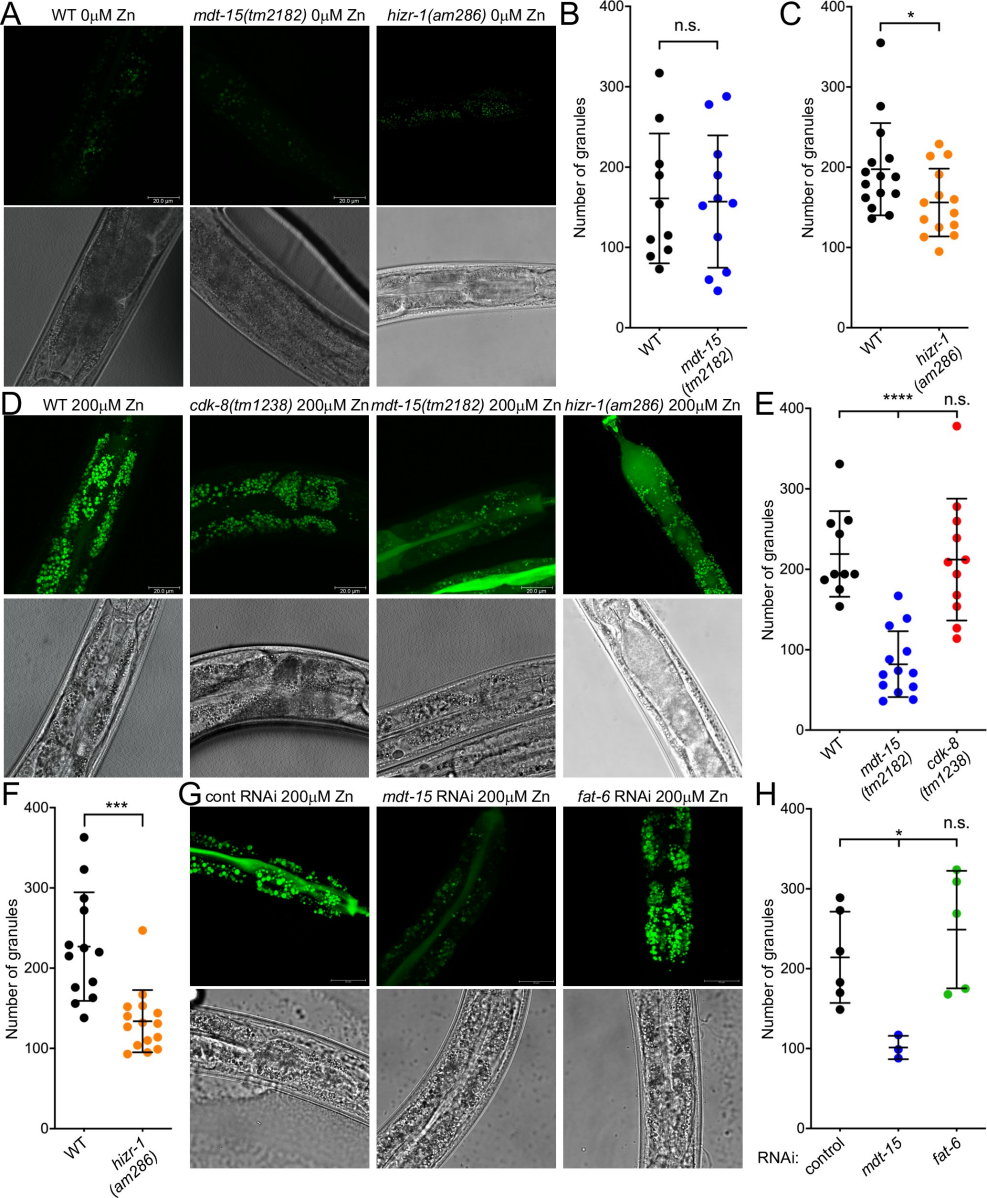
*mdt-15(tm2182)* mutants have a zinc storage defect. **[A]** Representative fluorescence and DIC micrographs of worms stained with FluoZin-3, showing zinc accumulation in the gut granules of wild-type (WT), *mdt-15(tm2182)*, and *hizr-1(am286)* worms grown without zinc supplementation. **[B-C]** Quantification of the number of gut granules in wild-type (WT), *mdt-15(tm2182)*, and *hizr-1(am286)* worms; every dot represents an individual worm. Error bars: standard deviation; n = 10 for WT in [B], 11 for *mdt-15(tm2182)*; 15 for WT in [C]; 14 for *hizr-1(am286)* (totals from 3 independent repeats for [B] and 4 for [C]). Statistical analysis: * p < 0.05, n.s. = not significant, unpaired Student’s t-test, comparisons to WT. **[D]** Representative fluorescence and DIC micrographs of worms stained with FluoZin-3, showing zinc accumulation in the gut granules of wild-type (WT), *mdt-15(tm2182)*, *cdk-8(tm1238)*, and *hizr-1(am286)* worms grown with 200μM zinc supplementation. **[E]** Quantification of the number of gut granules in wild-type (WT), *mdt-15(tm2182)*, and *cdk-8(tm1238)* worms; every dot represents an individual worm. Error bars: standard deviation; n = 10 for WT, 13 for *mdt-15(tm2182)*, 11 for *cdk-8(tm1238)* (totals from 3 independent repeats). Statistical analysis: **** p < 0.0001, One-way ANOVA, multiple comparisons, Dunnett correction, compared to “WT”. **[F]** Quantification of the number of gut granules in WT and *hizr-1(am286)* worms; every dot represents an individual worm. Error bars: standard deviation; n = 13 for WT, 15 for *hizr-1(am286)* (totals from 4 independent repeats). Statistical analysis: *** p < 0.001, unpaired Student’s t-test, compared to “WT”. **[G]** Representative fluorescence and DIC micrographs of worms fed control (empty vector L4440), *mdt-15*, or *fat-6* RNAi and stained with FluoZin-3, showing zinc accumulation in the gut granules of worms grown with 200mM zinc supplementation.**[H]** Quantification of the number of gut granules in worms fed control, *mdt-15*, or *fat-6* RNAi; every dot represents an individual worm. Error bars: standard deviation. n = 6 for control (2 independent repeats), 3 for *mdt-15* (1 repeat), and 5 for *fat-6* (2 independent repeats). Statistical analysis: * p < 0.05, n.s. = not significant, One-way ANOVA, multiple comparisons, Dunnett correction, compared to control RNAi.

To test whether the defects of *mdt-15(tm2182)* mutants in cadmium and zinc responsive transcription have functional consequences, we studied two phenotypes: cellular zinc storage and egg-laying. At the cellular level, gut granules store zinc when it is present in excess, thus protecting cells; *vice versa*, they replenish zinc in situations of zinc deficiency [50]. To study zinc storage in gut granules, we used the zinc-specific fluorescent dye FluoZin-3 [50]. We observed little difference between wild-type, *mdt-15(tm2182)*, *cdk-8(tm1238)*, and *hizr-1(am286)* mutants in “normal” conditions, i.e. without zinc supplementation (Fig 5A–5C). Wild-type worms supplemented with 200mM zinc showed bigger granules with a stronger FluoZin-3 signal (Fig 5D), as reported [50]. Strikingly, *mdt-15(tm2182)* mutants displayed significantly fewer gut granules in high zinc conditions than wild-type worms and *cdk-8(tm1238)* mutants (Fig 5D and 5E). As HIZR-1 interacts with MDT-15 in zinc-stimulated fashion, we also studied *hizr-1(am286)* null mutants and found that they also have less granules than wild-type worms in conditions of excess zinc (Fig 5D and 5F).

*mdt-15(tm2182)* mutants show reduced expression of several fatty acid metabolism enzymes, especially fatty acid desaturases such as *fat-6/stearoyl-CoA desaturase*. This causes defects in membrane fatty acid desaturation and directly underlies numerous phenotypes caused by *mdt-15* deficiency, including slow growth, reduced body size, and short life span [34,47,49,51,52]. Altered membrane lipids in *mdt-15(tm2182)* mutants could cause organelle dysfunction and conceivably affect zinc storage. Thus, we tested whether *fat-6/stearoyl-CoA desaturase* depletion by RNAi causes defects in zinc storage (note that *fat-6* RNAi also depletes the highly homologous gene *fat-7* [53]). In contrast to *mdt-15(tm2182)* mutants, however, *fat-6(RNAi)* worms did not show any overt defects in zinc storage in high zinc conditions (Fig 5G and 5H). This suggests that the zinc storage defects observed in the *mdt-15(tm2182)* mutants are not due to altered membrane composition and function.

**Fig 6:**
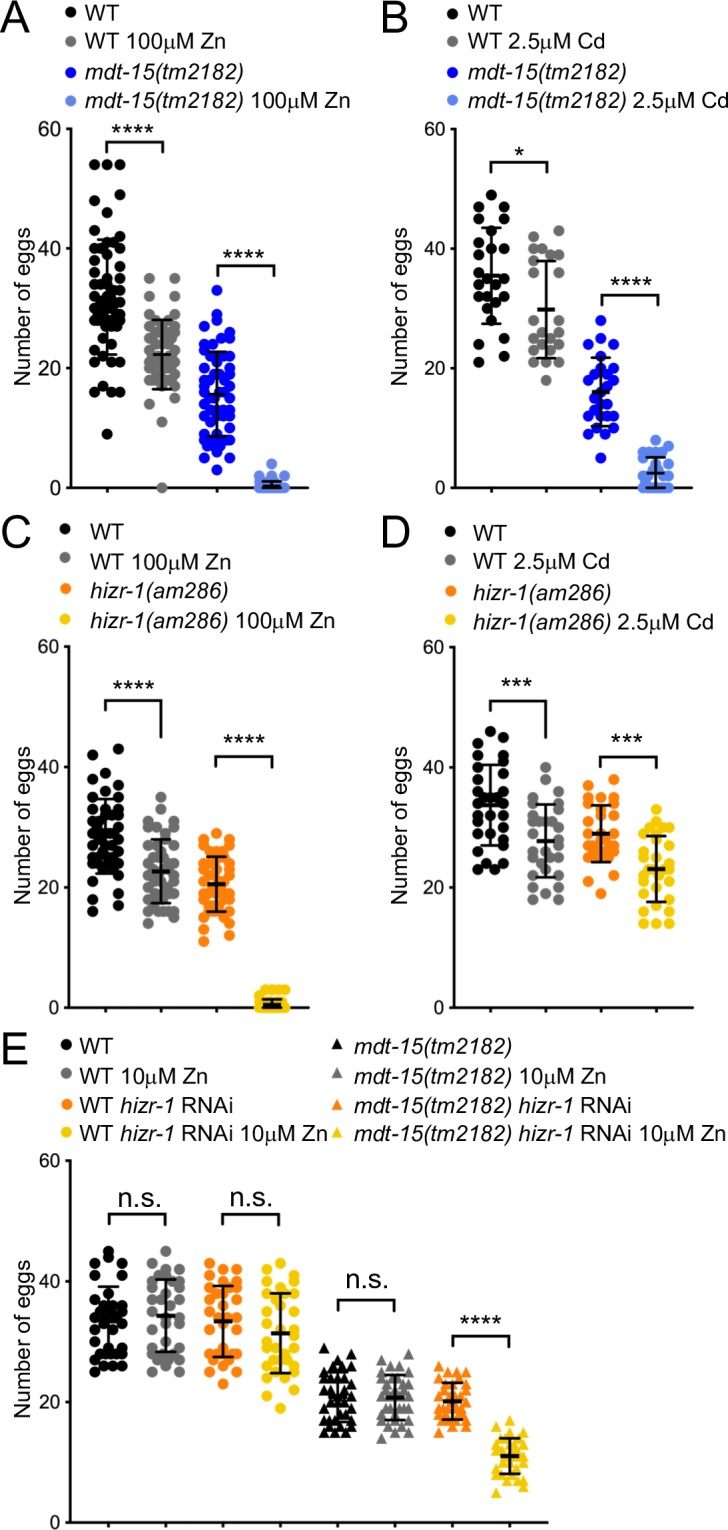
Loss of *mdt-15* renders worms sensitive to cadmium and zinc. **[A]** The graph shows the average number of eggs laid over a 24-hour period by WT worms and *mdt-15(tm2182)* mutants grown on 0μM and 100μM zinc (Zn). Each dot represents progeny from an individual worm (WT 0μM Zn: n = 59; WT 100μM Zn: n = 56; *mdt-15(tm2182)* 0μM Zn; n = 57; *mdt-15(tm2182)* Zn: n = 51; from 3 independent repeats). Error bars: standard deviation; statistical analysis: **** *p* < 0.0001, Two-way ANOVA, multiple comparisons; Tukey correction. **[B]** The graph shows the average number of eggs laid over a 24-hour period by WT worms and *mdt-15(tm2182)* mutants grown on 0μM and 2.5μM cadmium (Cd). Each dot represents progeny from an individual worm (WT 0μM Cd: n = 25; WT 2.5μM Cd: n = 23; *mdt-15(tm2182)* 0μM Cd: n = 26; *mdt-15(tm2182)* on 2.5μM Cd: n = 27; from 3 independent repeats). Error bars: standard deviation; statistical analysis: **** *p* < 0.0001, * *p* < 0.05, Two-way ANOVA, multiple comparisons; Tukey correction. **[C]** The graph shows the average number of eggs laid over a 24-hour period by WT worms and *hizr-1(am286)* mutants grown on 0μM and 100μM zinc (Zn). Each dot represents progeny from an individual worm (all groups n = 46; from 2 independent repeats). Error bars: standard deviation; statistical analysis: *** *p* < 0.001, **** *p* < 0.0001, Two-way ANOVA, multiple comparisons; Tukey correction. **[D]** The graph shows the average number of eggs laid over a 24-hour period by WT worms and *hizr-1(am286)* mutants grown on 0μM and 2.5μM cadmium (Cd). Each dot represents progeny from an individual worm (all groups n = 30; from 2 independent repeats). Error bars: standard deviation; statistical analysis: * *p* < 0.05, Two-way ANOVA, multiple comparisons; Tukey correction. **[E]** The graph shows the average number of eggs laid over a 24-hour period by WT worms and *mdt-15(tm2182)* mutants fed control or *hizr-1* RNAi and grown on 0μM and 10μM zinc (Zn). Each dot represents progeny from an individual worm (all groups n = 34; from 2 independent repeats). Error bars: standard deviation; statistical analysis: **** *p* < 0.0001, Two-way ANOVA, multiple comparisons; Tukey correction.

We also assessed the effect of excess zinc and cadmium on an organismal phenotype, egg-laying (Fig 6). We found that 100μM zinc decreased the number of eggs laid by wild-type worms by approximately 30 percent; in contrast, the same concentration of zinc almost completely abolished egg-laying in *mdt-15(tm2182)* mutants, indicating that this mutant is hyper-sensitive to zinc (Fig 6A). Similarly, 2.5μM cadmium reduced the number of eggs laid by wild-type worms by approximately 20 percent, but almost completely abrogated it in *mdt-15(tm2182)* mutants (Fig 6B), indicating that this mutant is hyper-sensitive to cadmium. Like the *mdt-15(tm2182)* mutant, the *hizr-1(am286)* null mutant also showed a virtually complete loss of egg-laying in 100μM zinc (Fig 6C), resembling the previously observed reduced growth of this mutant in conditions of high zinc [20]. However, the *hizr-1(am286)* mutant was not more sensitive to 2.5μM cadmium than WT worms in the egg-laying assay (Fig 6D). Thus, *mdt-15(tm2182)* mutant worms are less able than wild-type to resist excess zinc and cadmium at an organismal level, whereas the *hizr-1(am286)* mutant phenocopies the *mdt-15(tm2182)* mutant only in zinc, but not cadmium sensitivity.

Lastly, we used the egg-laying assay to study the genetic relationship between *mdt-15* and *hizr-1*. Specifically, we tested whether *hizr-1* RNAi would sensitize the *mdt-15(tm2182)* mutant’s egg-laying capacity in a low zinc concentration that doesn’t affect egg-laying on its own in either wild-type, *mdt-15(tm2182)*, or *hizr-1(RNAi)* worms. Interestingly, compared to control RNAi, *hizr-1* RNAi significantly reduced the number of eggs laid only in mutant worms and only in the presence of zinc, revealing a genetic interaction between *hizr-1* and *mdt-15* (Fig 6E). This suggests that the two genes may perform additional functions in zinc detoxification outside of their interaction in the same pathway.

### Mammalian MED15 is required for cadmium-induced transcription and binds the promoters of cadmium and zinc-induced genes

**Fig 7:**
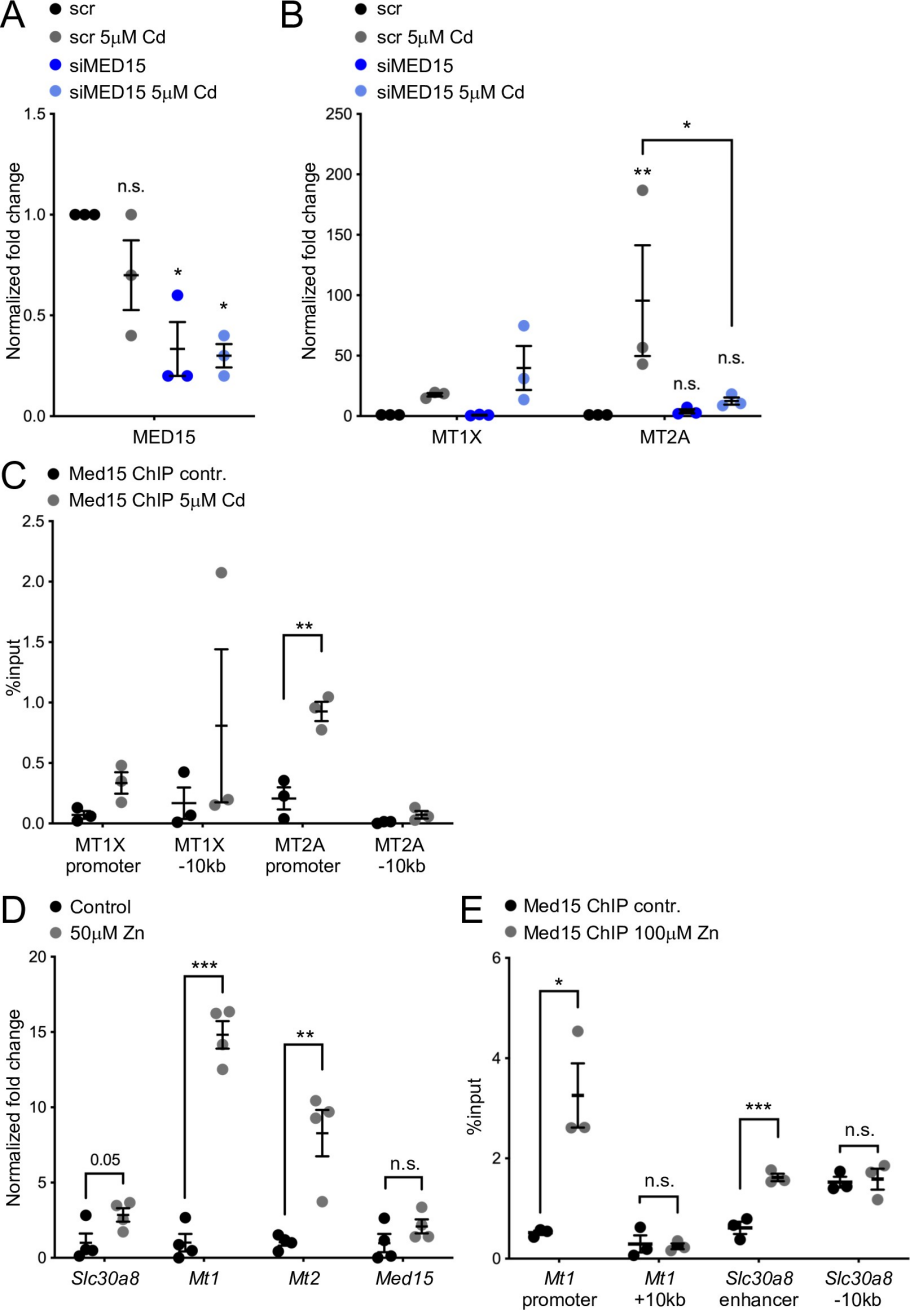
MED15 binds and regulates cadmium and zinc responsive genes in mammalian cells. **[A]** qRT-PCR analysis of MED15 mRNA in control (scr = scrambled siRNA) and MED15 siRNA (siMED15) treated A549 cells exposed to 5μM cadmium for four hours. Error bars: SEM; statistical analysis: * p < 0.05, One-way ANOVA, multiple comparison, Tukey correction, all comparisons to “scr” (n = 3 independent repeats). **[B]** qRT-PCR analysis of cadmium inducible genes in control (scr = scrambled siRNA) and MED15 siRNA (siMED15) treated A549 cells exposed to 5μM cadmium for four hours. Error bars: SEM; statistical analysis: * *p <* 0.05, ** *p* < 0.01, Two-way ANOVA, multiple comparisons, Tukey correction (n = 3 independent repeats). MT1X expression was not significantly affected. For MT2A, all comparisons are *vs*. “scr”, except where indicated by lines. **[C]** The graph shows relative MED15 occupancy at the MT1X and MT2A promoters and nearby control regions in A549 cells, as determined by ChIP-qPCR before and after the addition of 5μM cadmium. Error bars: SEM; statistical analysis: ** *p* < 0.01 Student’s t-test, multiple comparisons, Holm-Sidak correction (n = 3 independent repeats). **[D]** The graph shows qRT-PCR analysis of MIN6 cells treated without and with 50μM zinc. Error bars: SEM; statistical analysis: ** *p* < 0.01, *** *p* < 0.001 Student’s t-test, multiple comparisons, Holm-Sidak correction (n = 4 independent repeats). **[E]** The graph shows relative Med15 occupancy at the *Mt1* promoter and Slc30a8 enhancer and nearby control regions in MIN6 cells as determined by ChIP-qPCR, before and after the addition of 100μM Zn. Error bars: SEM; statistical analysis: * *p <* 0.05, *** *p* < 0.01 Student’s t-test, multiple comparisons, Holm-Sidak correction (n = 3 independent repeats).

To test whether *mdt-15*’s role in regulating metal-responsive transcription is conserved in mammalian cells we studied the role of its human and mouse orthologs, MED15 and Med15 [54], in two cell lines. First, we studied A549 human epithelial lung adenocarcinoma cells, because cadmium increases lung cancer risk and induces a stress response in this cell line [55–57]. We depleted MED15 in A549 cells by transfecting small interfering RNAs (siRNAs) targeting MED15 (*vs*. scrambled control), and then exposed the transfected cells to 5μM cadmium for 4 hours. MED15 siRNAs effectively knocked down MED15 as determined by qRT-PCR analysis (Fig 7A). Using qRT-PCR, we further found that cadmium induced the metallothioneins MT1X and MT2A, orthologues of *C*. *elegans mtl-1* and *-2*, respectively (Fig 7B). Notably, while MT1X was unaffected, MT2A induction was completely blocked by MED15 depletion (Fig 7B), suggesting that MED15 is required to induce this gene in response to cadmium exposure. To test whether MED15 binds to the promoter of MT2A, we performed chromatin immunoprecipitation followed by quantitative PCR (ChIP-qPCR), assessing MED15 occupancy before and after the addition of 5μM cadmium to the extracellular media. We found that MED15 was recruited preferentially to the promoter of MT2A, but not control regions, after addition of 5μM cadmium (Fig 7C).

We also studied the MIN6 mouse insulinoma cell line because the insulin-secreting β-cells of the mammalian pancreas require zinc for insulin crystallization and contain among the highest levels of zinc in the body [14,58,59]. ZnT8/SLC30A8, the mouse ortholog of the MDT-15 and HIZR-1 regulated zinc transporter CDF-2 [50], is expressed highly in the in α- and β-cells of the endocrine pancreas [14]. To test whether *Slc30a8* and metallothionein genes are induced by excess zinc, we exposed MIN6 cells to 50μM zinc for 24 hours and assessed expression by qRT-PCR. We observed that *Slc30a8*, *Mt1*, and *Mt2* are zinc responsive, whereas *Med15* is not (Fig 7D). To test whether Med15 directly binds to the promoters of *Slc30a8* and *Mt1*, we performed chromatin immunoprecipitation followed by ChIP-qPCR, assessing Med15 occupancy before and after the addition of excess zinc to the extracellular media. We found that Med15 was recruited to the promoters of both *Slc30a8* and *Mt1* in excess zinc (Fig 7E). Collectively, these experiments suggest that mammalian MED15 proteins directly bind the promoters of cadmium and zinc responsive genes and are required for the induction of at least one cadmium responsive gene in lung adenocarcinoma cells.

## Discussion

**Fig 8:**
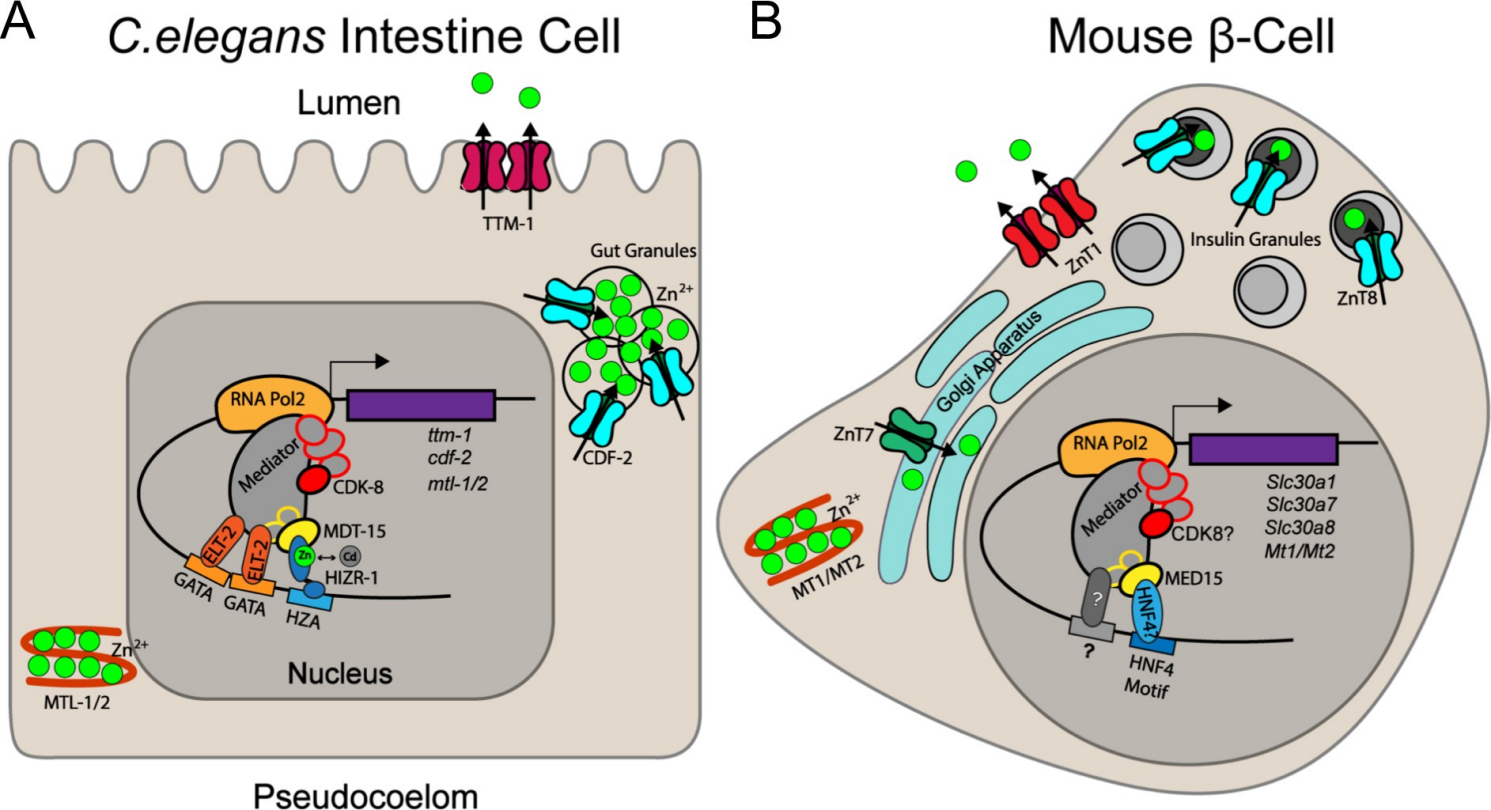
Model of MDT-15 and MED15 driven gene regulation in response to cadmium or high levels of zinc. **[A]** In the *C*. *elegans* intestinal cells, MDT-15 cooperates with HIZR-1 bound to the HZA element to induce the metal-sequestering metallothioneins (*mtl-1* and *-2*) and the transporter *cdf-2*, which shuttles zinc into storage granules; excess cadmium is likely detoxified through similar mechanisms. ELT-2 may also contact Mediator to regulate cadmium or zinc responsive transcription. **[B]** In the mouse pancreatic β-cells, Med15 regulates the expression of genes such as Slc30a8/ZnT8 in response to elevated zinc levels, possibly via HNF4, or other, Mediator contacting transcription factors.

Organisms encounter both essential and toxic metals in their habitant and must allow adequate uptake of necessary micronutrients while secreting/sequestering excess amounts of micronutrients and toxic metals. *C*. *elegans* lacks the MTF-1 protein that regulates metal responsive transcription in many animals. Instead, it utilizes the Nuclear Hormone Receptor HIZR-1 to control the response to excess zinc [20]. Here, we show that the *C*. *elegans* Mediator subunit MDT-15 interacts physically and functionally with HIZR-1, that loss of *mdt-15* or *hizr-1* alters storage of excess zinc *in vivo*, and that *mdt-15* mutants are hypersensitive to zinc and cadmium. Moreover, mammalian MED15 is recruited to regulatory elements of cadmium and zinc-responsive genes in metal-stimulated fashion, and MED15 depletion blocks the induction of a cadmium responsive gene in lung adenocarcinoma cells. Thus, the HZA element, HIZR-1 transcription factor, and MDT-15 coregulator compose a regulatory mechanism that adapts gene expression in response to excess zinc and cadmium to protect the host organism (Fig 8A). This regulatory mechanism represents a new partnership between a *C*. *elegans* NHR and the coregulator MDT-15 that regulates one particular adaptive response, that to cadmium and excess zinc. Moreover, this response mechanism is at least partly conserved in mammalian cells (Fig 8B).

### MDT-15 is a coregulator of HIZR-1

Because *C*. *elegans* appears to lack an MTF-1 ortholog, it has been unclear how it regulates gene expression in response to changing levels of various metals. We previously showed that the Mediator subunit *mdt-15* is essential for both zinc and cadmium activated transcription [33], and the Kornfeld laboratory identified the HZA element and its cognate TF HIZR-1 as regulators of zinc-inducible transcription [19,20]. This raised the hypothesis that these three components might all cooperate mechanistically; alternatively, they might act in separate molecular pathways. Here, we provide multiple lines of evidence that HIZR-1 and MDT-15 cooperate mechanistically to induce gene expression in excess zinc and cadmium. First, *mdt-15*, *hizr-1*, and the HZA element are required to activate the *cdr-1p*::*gfp* reporter, i.e. they phenocopy (Figs 2 and 3; see also [19,20]). Second, MDT-15 and HIZR-1 physically interact in the yeast-two-hybrid system; notably, this binding can be stimulated by a known ligand of HIZR-1, zinc [20], and requires a known NHR binding domain of MDT-15, the KIX-domain [34,35] (Fig 4). Third, genetic gain of *hizr-1* or *mdt-15* function confers metal homeostasis gene activation that requires the reciprocal partner (Fig 4). Fourth, loss of *mdt-15* and *hizr-1* causes similar defects in zinc storage and renders worms hypersensitive to zinc (Figs 5 and 6 and [20]). We cannot exclude that ELT-2, which activates zinc responsive genes, including *cdr-1*, and binds the GATA DNA element, a general feature of zinc-responsive promoters (Fig 3, [19]), may interact with MDT-15; however, we have not observed an interaction between ELT-2 and MDT-15 in our Y2H assays. Based on our new and published data, we propose a model whereby elevated levels of zinc or cadmium promote the formation of a HIZR-1–MDT-15 regulatory complex, which acts through the HZA element to induce the expression of genes required for zinc homeostasis and cadmium detoxification (Fig 8).

### MDT-15 binds HIZR-1 in zinc and cadmium-stimulated fashion in yeast two hybrid assays

Interestingly, zinc or cadmium supplementation enhanced MDT-15–HIZR-1 binding in our Y2H assays (Fig 4). This suggests that, besides promoting HIZR-1 nuclear translocation [20], these metals also modulate the TF-Mediator interaction, a classical feature of *bona fide* NHR ligands [60]. Importantly, two other HNF4-related NHRs that also bind MDT-15 [34] did not show cadmium, zinc, or manganese-enhanced binding. This agrees with the notion that zinc is a specific ligand of HIZR-1 [20] and is not involved in the MDT-15–NHR interactions through the zinc-finger containing DBDs of NHRs [21].

In the Y2H system, we observed increased binding between HIZR-1 and MDT-15 in the low micromolar range of zinc and cadmium. We acknowledge the possibility that these metals could indirectly increase β-galactosidase activity, used here as a readout of interaction strength, e.g. by affecting positioning of the Gal4 activation domain for activation, multimerization, or better stability of the GAD fusion (although we did not observe altered expression of GAD fusion proteins, S1 Fig). However, while we acknowledge that our studies were performed in this heterologous host and system, the concentration of zinc required to modulate the HIZR-1–MDT-15 interaction resembles that known to engage other zinc homeostasis proteins. Specifically, murine ZIP4 undergoes zinc-stimulated endocytosis when zinc is present in the low micromolar range [61], and various human and mouse ZIP proteins promote zinc transport in the low micromolar range [62]. This supports the notion that the zinc-modulation of the protein interaction we observed in the Y2H system is likely relevant physiologically.

Our transcriptome comparison (Fig 1) suggested that, besides MDT-15, CDK-8 might also be required to activate cadmium responsive genes, including *cdr-1*. Hence, we hypothesized that MDT-15 and CDK-8 perform similar roles in in cadmium and zinc detoxification, perhaps cooperating in this process. Such cooperation would be distinct from the functional antagonism we observed for MDT-15 and CDK-8 in vulva development, wherein *cdk-8* appears to restrain MDT-15 activity and downregulate its protein levels [38], resembling the antagonism observed for yeast Med15 and Cdk8 [39]. Our follow-up studies suggested that *cdk-8* affects only two cadmium and zinc responsive genes and does not impact zinc storage in high zinc conditions (Figs 1, 2 and 5). Hence, CDK-8’s role appears more limited than that of MDT-15 and we did not pursue it further. Individual Mediator subunits are known to play specific roles and display differential requirements in in developmental and physiological contexts, so the comparably minor role for *cdk-8* is not unexpected. Nevertheless, it is possible that other physical interactions between transcription factors and Mediator subunits contribute to the zinc and cadmium homeostasis mechanism we describe herein, perhaps linking HIZR-1 or MDT-15 to ELT-2 or CDK-8 (Fig 8). In addition, HNF4-like NHRs form homo- and heterodimers [21,63]. It would be interesting to examine whether HIZR-1 forms homodimers or heterodimers with other NHRs, and whether such putative interactions are modulated by zinc and/or cadmium.

### *mdt-15* protects worms from excess zinc and cadmium

In the *C*. *elegans* intestine, zinc is stored in lysosome-related organelles called gut granules. In conditions of excess zinc, gut granules import surplus zinc via the CDF-2 transporter [50], while zinc is likely also sequestered by MTL-1 and -2. MDT-15 and HIZR-1 promote the induction of these genes when zinc is in excess. This induction is likely physiologically relevant as mutation of either factor results in reduced zinc storage in gut granules and reduced organismal zinc and cadmium tolerance (Figs 5 and 6 and [20]).

The study that established zinc as a direct ligand for HIZR-1 did not test whether this NHR also bound cadmium [20]. Our data suggest that this may be the case, as cadmium stimulates MDT-15–HIZR-1 binding as strongly as does zinc in our Y2H assays (Fig 4), and *mdt-15* mutants were sensitive to both metals (Figs 5 and 6). However, we note that cadmium could also act indirectly by displacing zinc from other molecular sites and thus making it available for HIZR-1, resulting in higher apparent zinc levels that indirectly engage the MDT-15–HIZR-1 complex. It will be interesting to test whether HIZR-1 is indeed a direct sensor of the pollutant cadmium.

MDT-15 also promotes oxidative stress responses [36,37,48] and it is conceivable that this function helps protect worms in conditions of excess cadmium, which provokes the formation of reactive oxygen species [7,55]. It will be interesting to determine whether HIZR-1 regulates genes other than those involved in storing and mobilizing zinc, such as general or oxidative stress protective genes.

### MDT-15 and HIZR-1 may also perform additional, independent functions

Our physical and genetic interaction studies support the view that MDT-15 and HIZR-1 cooperate in a genetic pathway for high zinc and cadmium detoxification. However, two pieces of data suggest that the proteins may also act independently in this context. First, although *hizr-1* depletion abrogates *cdr-1* induction by cadmium (Fig 3A), the *hizr-1* null mutant was not sensitive to cadmium in our egg-laying assay (Fig 6D); in contrast, *mdt-15* mutant worms were highly sensitive to cadmium in this assay (Fig 6B). These data suggest that MDT-15 may cooperate with additional TFs in the regulation of cadmium responsive genes and show that cadmium and zinc detoxification are not identical processes. Second, in a genetic interaction study, we found that combined inactivation of *hizr-1* and *mdt-15* resulted in synthetic sensitivity to low levels of zinc (Fig 6E). A limitation of this experiment is the use of a hypomorphic allele for *mdt-15* and of RNAi for *hizr-1*. However, collectively, these two experiments show that that these two regulatory factors likely perform additional, independent functions that will be interesting to dissect in future studies.

### Mammalian MED15 also regulates cadmium and zinc responsive gene transcription

*C*. *elegans* MDT-15 plays an important role in many metabolic and stress response pathways. Of those, its role in regulating lipid metabolism appears to be conserved in yeast and in mammals [49,64], whereas its requirement in detoxifying xenobiotic molecules is conserved in fungi [28,65]. However, whether mammalian MED15 proteins regulate stress responses was not known to date. Studying a lung adenocarcinoma cell line that responds to cadmium, we found that MED15 depletion compromises the induction of a metallothionein and that MED15 directly binds the genomic regulatory region of this gene in cadmium enhanced fashion. Similarly, mouse Med15 showed zinc induced binding to the regulatory regions of two genes in MIN6 insulinoma cells; although we did not succeed in effectively depleting Med15 in these cells with transfected siRNAs, we found that these genes are induced by zinc. Thus, we speculate that the increased binding of Med15 likely upregulates their expression in high zinc. In turn, this likely promotes the protection of MIN6 cells from high zinc (Fig 8B). A similar mechanism may protect pancreatic islet β-cells from the transiently high zinc levels these cell experience during insulin exocytosis [58,59].

Currently, we do not know what transcription factor cooperates with mammalian MED15 proteins in cadmium and/or zinc responsive gene expression. MTF-1 induces cadmium responsive genes in mammals and interacts with Mediator [17], although it is not known which, if any, Mediator subunit directly targets MTF-1. *C*. *elegans* HIZR-1 is by sequence most closely related to mammalian HNF4α, but functionally and structurally may also resemble mammalian PPARα [21,51]. In mouse livers, exposure to the PPARα agonist Wy-14,643 induces *Mt1* and *Mt2* mRNA levels; however, induction is modest (approximately 1.8 fold) and delayed (after 72 hours) [66]. Thus, this may well represent an indirect regulatory effect. Additional work is required to determine the mechanisms by which Mediator, and potentially MED15, regulate gene expression in response to high concentrations of metals in mammalian cells and tissues.

In sum, our work highlights the HZA–HIZR-1–MDT-15 regulatory mechanism as a critical transcriptional adaptive mechanism to excess zinc and cadmium in *C*. *elegans*, with a partially conserved role for mammalian MED15.

## Materials and methods

### *C*. *elegans* transcriptome analysis by microarrays

Microarray transcriptome analysis of the *cdk-8(tm1238)* mutant has been described [38]. The transcriptome analysis of the *mdt-15(tm2182)* mutant was identical to the analysis of the *cdk-8(tm1238)* mutant using Agilent one-color arrays. We identified a total of 1896 spots with an adjusted P-value of 0.05 or less and a fold-change of >2, representing 798 downregulated and 422 upregulated genes. Microarray data have been deposited in Gene Expression Omnibus. Transcriptome profiles of wild-type worms exposed to 100μM cadmium have been described [43]. We determined the overlaps between these datasets and calculated the significance of the overlap as described [36]. A list of genes in these overlaps is provide in S1 Table.

### *C*. *elegans* strains and growth conditions

*C*. *elegans* strains were cultured using standard techniques as described [67] at 20°C; all strains used in this study are listed in S2 Table. Nematode growth medium (NGM)-lite (0.2% NaCl, 0.4% tryptone, 0.3% KH_2_PO_4_, 0.05% K_2_HPO_4_) agar plates, supplemented with 5μg/mL cholesterol, were used unless otherwise indicated. *Escherichia coli* OP50 was the food source, except for RNAi, for which we used HT115. Zinc (ZnSO_4_) and cadmium (CdCl_2_) were supplemented in noble agar minimal media (NAMM; [50]) and NGM-lite plates, respectively, at indicated concentrations. For qRT-PCR and phenotype analysis, we synchronized worms by standard sodium hypochlorite treatment and starvation of isolated eggs on unseeded NGM-lite plates; the next day, synchronized L1 larvae were collected, placed on seeded plates at the desired densities, and grown until being harvested at the desired developmental stage, as indicated.

### Gene knockdown by feeding RNAi in *C*. *elegans*

Knockdown by feeding RNAi was carried out on NGM-lite plates with 25μg/mL carbenicillin, 1mM IPTG, and 12.5μg/mL tetracycline. Plates were seeded twice with the appropriate HT115 RNAi bacteria clone from the Ahringer library (Source BioScience 3318_Cel_RNAi_complete). RNAi clones were Sanger sequenced to confirm insert identity. RNAi negative control was empty vector L4440. RNAi clones are listed in S3 Table.

### RNA isolation and quantitative real-time PCR analysis

For *C*. *elegans*, RNA was extracted from developmentally synchronized worms and prepared for gene expression analysis by real-time PCR analysis as described [38]. In all samples, we normalized the expression of the tested genes to the average of three normalization genes: *act-1*, *tba-1*, and *ubc-2*. For A549 and MIN6 cells, RNA was extracted with RNeasy Mini Kit (Qiagen #74106) according to the manufacturer’s protocol and converted into cDNA for gene expression analysis by real-time PCR analysis as described [38]. The expression of the tested genes was normalized using the following normalization genes: 18S rRNA, GAPDH, and/or GUSB. We used t-tests (two-tailed, equal variance) or nonparametric tests to calculate the significance of expression changes between conditions, as indicated. Statistical tests were performed based on recommendations by GraphPad Prism 7. All qRT-PCR reactions were performed in technical triplicates; for number of independent biological experiments done, see figure legends. qRT-PCR primers were designed with Primer3web [68] and tested on serial cDNA dilutions, as described [38]. Primer sequences are listed in S4 Table.

### Analysis of the *C*. *elegans cdr-1* promoter and construction of the *cdr-1p*::*gfp* promoter reporter

Sequences of regulatory elements involved in the zinc, cadmium, or other detoxification/stress responses were identified from the literature, including ARE, MRE, HSE, DBE, HZA, GATA, and TATA box elements [19,69–78]. If available, the corresponding *C*. *elegans* consensus sequence for each regulatory element was identified in the literature, otherwise the eukaryotic consensus sequence was used. The *cdr-1* promoter (-2853 nucleotides upstream from the predicted transcriptional starting site) was then searched for presence of these candidate elements using SerialCloner 2.5.

The *cdr-1p*::*gfp* reporter was generated by PCR amplification of the genomic region from 2853 base pairs upstream to 11 base pairs downstream of the *cdr-1* start codon (a G>C mutation at the +3 nucleotide was introduced in the reverse primer to mutate the *cdr-1* start codon) using the primers gtcgacTTTGACGATGACAGAAGAAATG and ggatccTGAATCCAAGATACTTGAGACAGT, followed by BamH1-SalI cloning into pPD95.77 GFP (Addgene #1495) to generate SPD771 (cdr-1p-pPD95.77). Mutant transgenes were generated by site-directed mutagenesis of SPD771 using the Q5 Site-Directed Mutagenesis kit (NEB E0554S) and primers cdr-1p_delGATA1F (CCCTACTTTCccgctgCATTATGTCATCGGG) and cdr-1p_delGATA1R (GTTTCTGTTTCAATTGCAGAATAC) to generate SPD803 (cdr-1p-pPD95.77_DEL_GATA1); cdr-1p_delGATA2F (CCCTACTTTCccgctgCATTATGTCATCGGG) and cdr-1p_delGATA2R (AGAACTGTGTTTTGTGATAAAATTATTG) to generate SPD804 (cdr-1p-pPD95.77_DEL_GATA2); and fwd_cdr-1p_delHZA (tcgtggcAATTTTATCACAAAACACAGTTC) and rev_cdr-1p_delHZA (ccctcaccTCAATTGCAGAATACCATTTG) to generate SPD884 (pPD95.77-cdr-1PmutHZA). All plasmids were verified by Sanger sequencing. Transgenic strains were generated by injecting a mixture of 50 ng/μl GFP reporter plasmid, 5 ng/μl pCFJ90*[myo-2p*::*mCherry]*, and 95 ng/μl pPD95.77 empty vector into wild-type worms, and then selecting transgenic mCherry-positive progeny.

### Staining of *C*. *elegans* gut granules with FluoZin-3

FluoZin-3 acetoxymethyl ester (Molecular Probes F24195) was reconstituted in dimethylsulfoxide (DMSO) to a 1mM stock solution. FluoZin-3 was diluted in M9 to generate a concentration of 30μM and dispensed on NAMM plates, as described [50]. Synchronized wild-type and mutant L4 stage worms were transferred from NGM-lite plates to these plates and cultured for 16 hrs. Worms were then transferred to NGM-lite plates without FluoZin-3 for 30 min to reduce excess FluoZin-3 signal from the intestinal lumen before imaging.

### Fluorescence microscopy on *C*. *elegans*

For imaging, worms were transferred onto 2% (w/v) agarose pads containing 15μM sodium azide (NaN_3_; Sigma). For the analysis of worms bearing the *cdr-1p*::*gfp* reporter, the worms were imaged using differential interference contrast (DIC) and fluorescence optics through an HQ camera (Photometrics, Tucson, AZ, USA) on a Zeiss Axioplan 2 microscope (Carl Zeiss Microscopy, Thornwood, NY, USA). Analysis of fluorescence intensity was performed using ImageJ software, normalizing for area and background fluorescence.

For analysis of FluoZin-3 stained worms, we used a Leica SP8 confocal microscope with Leica LAS X software. Images of worms with different genotypes were taken with the same exposure times. To assess zinc storage, we quantified the number of FluoZin-3 stained granules in the first six cells of the gut with ImageJ2 [79]. To eliminate non-specific fluorescence, we manually removed background signal outside of the gut cells and in the gut lumen. Background fluorescence in the remaining part of the image was then subtracted equally from all images, and images were smoothed with the “Sigma Filter Plus” (edge-preserving noise reduction) function. Images were simultaneously adjusted with Auto threshold of “MaxEntropy” and Auto local threshold of the mean. After adjusting the threshold and reducing background noise, granules where counted automatically with the “3D Objects Counter” function. To verify the automatic count, we manually counted the number of granules in randomly sampled images.

### *C*. *elegans* egg laying assay

N2 and *mdt-15(tm2182)* worms were grown from late L4 stage for 24 hours on agar A plates seeded with OP50 and supplemented with 100μM zinc or 2.5μM cadmium. Eggs and L1 progeny were counted after that time and compared to worms grown on agar A plates with no additional treatment.

### Yeast-two-hybrid assays and Western blots

MDT-15 bait plasmids and NHR-49, NHR-64, and SKN-1c prey plasmids have been described [48]. The wild-type HIZR-1 cDNA sequence was amplified with the primers fwd_BamHI_NHR-33_preyY2H (ggatccATGCAAAAAGTTATGAATGATCCTG) and rev_XhoI_NHR-33_preyY2H (ctcgagATCATTTTTCGTATGAACAATGCAC), and cloned into the BamH1 and SalI sites of pGADnewMCS to generate SPD885 (pGADnewMCS_NHR-33). We then used the NEB Q5 site-directed mutagenesis kit (E0554S), template SPD885, and primers SDM_nhr-33-am285_F (AGCAGAAaATGCTGCAAAAAT) and SDM_nhr-33-am285_R (GATTGTACACCCTCTGCATTC) to generate SPD918 (pGADnewMCS_NHR-33_am285). All plasmids were sequenced to verify the accuracy of the sequence amplified by PCR and the absence of other mutations. Pairs of plasmids were transformed into strain Y187 (Clontech, Mountain View, CA, USA) and liquid β-galactosidase assays were performed using an OMEGASTAR plate reader (BMG Labtech, Ortenberg, Germany), as described [48]. Each assay included at least three technical replicates; for number of independent biological experiments done, see figure legends. Yeast lysis, SDS-PAGE, and Western blots to detect protein expression were done as described [48]. Antibodies used were GAL4 AD Mouse Monoclonal Antibody (Takara Bio USA, Inc. #630402) and GAPDH Mouse Monoclonal Antibody (CB1001-500UG 6C5) for normalization.

### Mammalian cell culture and transfection

A549 lung adenocarcinoma cells were obtained from ATCC and maintained in Dulbecco's Modified Eagle Medium (DMEM; Gibco #11995065) supplemented with 10% fetal bovine serum (FBS; Gibco #12484028), as described [57]. The cells were seeded at a density of 5x10^4^ cells per well in 24-well plates 24 hours before transfection. On the day of transfection, scrambled (Dharmacon #D-001206-13-05) and MED15 specific (Dharmacon #M-017015-02-0005) siRNAs were delivered into A549 cells using DharmaFECT 1 Transfection Reagent (Dharmacon #T-2001-03) according to the manufacturer’s protocol. The transfection medium was replaced with complete medium after 24 hours, and the cells were treated with 5μM CdCl_2_ for hours 48 hours post-transfection. MIN6 cells were cultured in 25mM glucose DMEM, as described [80], and zinc stimulation was performed by addition of 50μM ZnSO_4_ for 24 hours.

### ChIP in A549 and MIN6 cells

ChIP was performed as described [81] in A549 and MIN6 cells with minor modifications. Briefly, cells were grown for 24 hours with or without 50μM CdCl_2_ and 100μM ZnSO_4_ (respectively) to 2x10^7^ cells per plate, and crosslinked by adding paraformaldehyde to a final concentration of 3% for 10 minutes at room temperature. Immunoprecipitation was performed using MED15 antibody (ProteinTech, 11566-1-AP). Crosslinking was then reversed by overnight incubation at 65^°^C and DNA purified using QIAquick PCR purification column (Qiagen, 28104). Immunoprecipitated DNA was then quantified via Qubit (ThermoFisher, Q32854) and analyzed by qPCR for appropriate genomic regulatory loci and controls. Primer sequences are listed in S4 Table.

### Raw data

All numerical values used for figures in this study are provided in S5 Table.

## Supporting information

S1 Fig Expression analysis of fusion proteins used in Y2H assays. Expression of Y2H prey fusion proteins was assessed by immunoblot with Gal4 Activation Domain antibody, with GAPDH serving as loading control. **[A]** Expression of proteins for assay in Fig 4C. **[B]** Expression of proteins for assay in Fig 4D. **[C]** Expression of proteins for assay in Fig 4E. **[D]** Expression of proteins for assay in Fig 4G. **[E]** Expression of proteins for assay in Fig 4H. These blots represent one of multiple independent repeats that were averaged to generate the data in the Fig 4 (n = 3–4, see Fig 4 legend for detail).
(TIF)

S2 Fig Conservation of the D270 residue in HIZR-1 across species. Partial alignments (generated with Clustal Omega) of the protein sequences of HIZR-1 homologues from *C*. *elegans* (WormBase ID: CE43818), *C*. *briggsae* (WormBase ID: CBP40955), *C*. *brennerei* (WormBase ID: CN15900), *C*. *japonica* (WormBase ID: JA59124), *C*. *remanei* (WormBase ID: RP48667), and *Pristionchus pacificus* (WormBase ID: PP70359). The black arrow indicates the D270 residue affected by the *am285* gf mutation in *C*. *elegans hizr-1*.
(TIF)

S1 Table Overlap of genes downregulated in *cdk-8(tm1238)* mutants and/or *mdt-15(tm2182)* mutants with genes induced by cadmium. (XLSX)

S2 Table List of worm strains used in this study. (DOCX)

S3 Table List of HT115 RNAi bacteria clones from the Ahringer library. (DOCX)

S4 Table List of primers used in qRT-PCR and ChIP-qPCR experiments. (DOCX)

S5 Table Numerical values used for figures in this study. (XLSX)
