# Supplementary figures and images for: Mediator subunit MDT-15/MED15 and Nuclear Receptor HIZR-1/HNF4 cooperate to regulate toxic metal stress responses in Caenorhabditis elegans
Source: PLoS Genet. 2019 Dec 9;15(12):e1008508. doi: 10.1371/journal.pgen.1008508 (PMC6922464; doi:10.1371/journal.pgen.1008508)

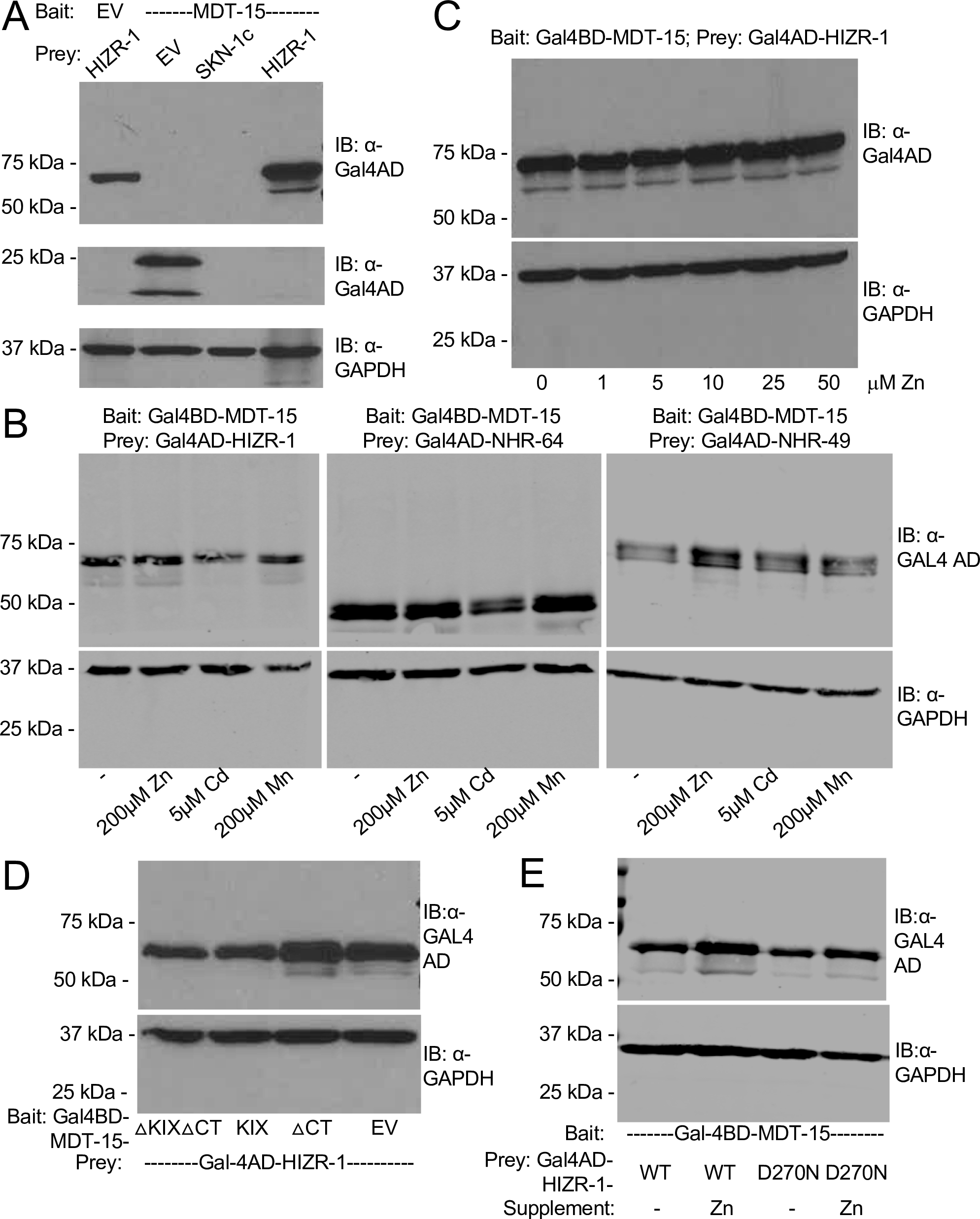

Supplement: S1 Fig — Expression of Y2H prey fusion proteins was assessed by immunoblot with Gal4 Activation Domain antibody, with GAPDH serving as loading control. [A] Expression of proteins for assay in Fig 4C. [B] Expression of proteins for assay in Fig 4D. [C] Expression of proteins for assay in Fig 4E. [D] Expression of proteins for assay in Fig 4G. [E] Expression of proteins for assay in Fig 4H. These blots represent one of multiple independent repeats that were averaged to generate the data in the Fig 4 (n = 3–4, see Fig 4 legend for detail). (TIF) [file pgen.1008508.s001.tif]

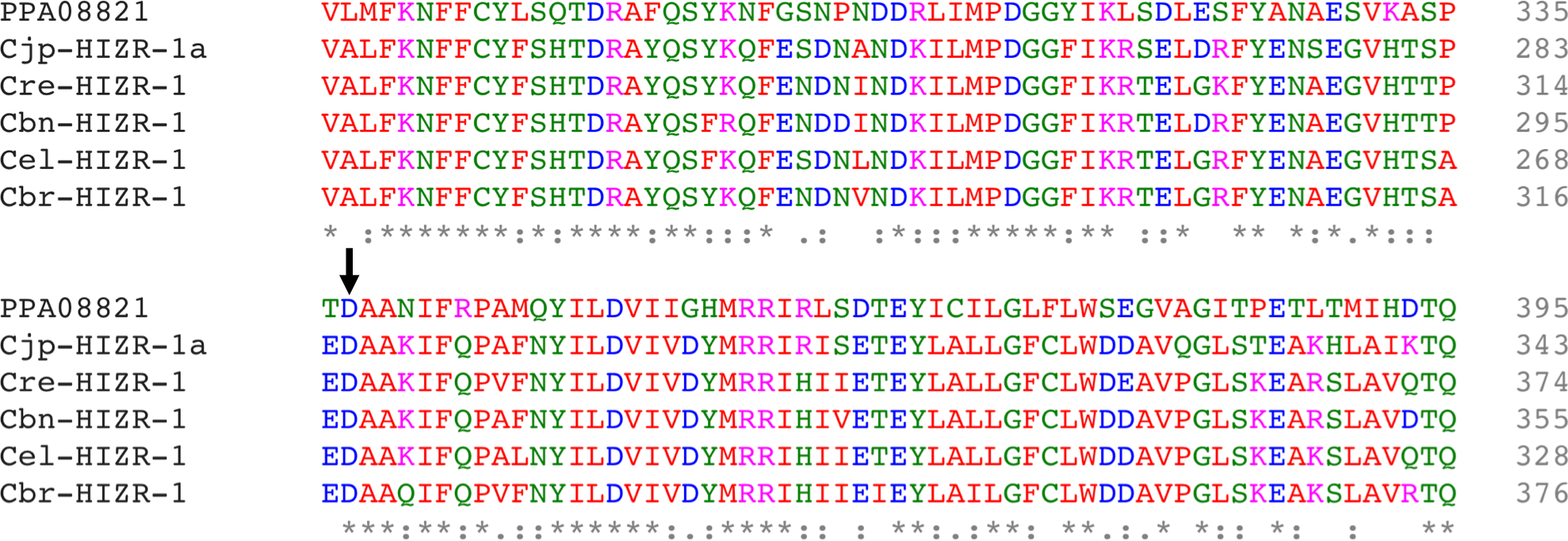

Supplement: S2 Fig — Partial alignments (generated with Clustal Omega) of the protein sequences of HIZR-1 homologues from C. elegans (WormBase ID: CE43818), C. briggsae (WormBase ID: CBP40955), C. brennerei (WormBase ID: CN15900), C. japonica (WormBase ID: JA59124), C. remanei (WormBase ID: RP48667), and Pristionchus pacificus (WormBase ID: PP70359). The black arrow indicates the D270 residue affected by the am285 gf mutation in C. elegans hizr-1. (TIF) [file pgen.1008508.s002.tif]
