# Supplementary material for: Mediator subunit MDT-15/MED15 and Nuclear Receptor HIZR-1/HNF4 cooperate to regulate toxic metal stress responses in Caenorhabditis elegans
Source: PLoS Genet. 2019 Dec 9;15(12):e1008508. doi: 10.1371/journal.pgen.1008508 (PMC6922464; doi:10.1371/journal.pgen.1008508)
Supplement: S2 Table — (DOCX) [file pgen.1008508.s004.docx]

**S2 Table: List of worm strains used in this study.**

| Strain | Strain name | Reference |
| --- | --- | --- |
| Wild type | N2 | [1] |
| *mdt-15(tm2182) III* | XA7702 | [2] |
| *cdk-8(tm1238) I* | XA7703 | [3] |
| *steEx49[cdr-1P::GFP + myo-2P::mCherry]* | STE101 | This study |
| *cdk-8(tm1238) I; steEx49[cdr-1P::GFP + myo-2P::mCherry]* | STE102 | This study |
| *steEx50[cdr-1P(mutHZA)::GFP + myo-2P::mCherry]* | STE103 | This study |
| *steEx51[cdr-1P(mutGATA1)::GFP + myo-2P::mCherry]* | STE104 | This study |
| *steEx52[cdr-1P(mutGATA2)::GFP + myo-2P::mCherry]* | STE105 | This study |
| *steEx56[cdr-1P(mGATA1,2)::GFP + myo-2P::mCherry]* | STE121 | This study |
| *hizr-1(am285) X* | WU1500 | [4] |
| *hizr-1(am286) X* | WU1563 | [4] |
| *mdt-15(yh8) III* | IJ1467 | [5] |

**References**

1. Brenner S. The genetics of Caenorhabditis elegans. Genetics. 1974;77: 71–94.

2. Taubert S, Hansen M, Van Gilst MR, Cooper SB, Yamamoto KR. The Mediator subunit MDT-15 confers metabolic adaptation to ingested material. PLoS Genet. 2008;4: e1000021. doi:10.1371/journal.pgen.1000021

3. Grants JM, Ying LTL, Yoda A, You CC, Okano H, Sawa H, et al. The Mediator Kinase Module Restrains Epidermal Growth Factor Receptor Signaling and Represses Vulval Cell Fate Specification in Caenorhabditis elegans. Genetics. 2016;202: 583–599. doi:10.1534/genetics.115.180265

4. Warnhoff K, Roh HC, Kocsisova Z, Tan C-H, Morrison A, Croswell D, et al. The Nuclear Receptor HIZR-1 Uses Zinc as a Ligand to Mediate Homeostasis in Response to High Zinc. PLoS Biol. 2017;15: e2000094. doi:10.1371/journal.pbio.2000094

5. Lee D, An SWA, Jung Y, Yamaoka Y, Ryu Y, Goh GYS, et al. MDT-15/MED15 permits longevity at low temperature via enhancing lipidostasis and proteostasis. PLoS Biol. 2019;17: e3000415. doi:10.1371/journal.pbio.3000415
