# Supplementary material for: Mediator subunit MDT-15/MED15 and Nuclear Receptor HIZR-1/HNF4 cooperate to regulate toxic metal stress responses in Caenorhabditis elegans
Source: PLoS Genet. 2019 Dec 9;15(12):e1008508. doi: 10.1371/journal.pgen.1008508 (PMC6922464; doi:10.1371/journal.pgen.1008508)
Supplement: S3 Table — (DOCX) [file pgen.1008508.s005.docx]

**S3 Table: List of HT115 RNAi bacteria clones from the Ahringer library.**

| Gene name | Sequence name | Plate & well number |
| --- | --- | --- |
| *skn-1* | T19E7.2 | 99 G09 |
| *hsf-1* | Y53C10A.12 | 21 B05 |
| *daf-16* | R13H8.1 | 18 G12 |
| *hizr-1* | ZK455.6 | 193 H07 |
| *elt-2* | C33D3.1 | 192 E06 |
| *mdt-15* | R12B2.5 | 74 C09 |
| *fat-6* | VZK822L.1 | 113 D11 |
