# Supplementary material for: Mediator subunit MDT-15/MED15 and Nuclear Receptor HIZR-1/HNF4 cooperate to regulate toxic metal stress responses in Caenorhabditis elegans
Source: PLoS Genet. 2019 Dec 9;15(12):e1008508. doi: 10.1371/journal.pgen.1008508 (PMC6922464; doi:10.1371/journal.pgen.1008508)
Supplement: S4 Table — (DOCX) [file pgen.1008508.s006.docx]

**S4 Table: List of primers used in qRT-PCR and ChIP-qPCR experiments.**

| Gene | Forward primer | Reverse primer |
| --- | --- | --- |
| mRNA expression analysis | | |
| *mtl-1* | TGGATGTAAGGGAGACTGCAA | CATTTTAATGAGCCGCAGCA |
| *mtl-2* | AAGTGTGCCAACTGCGAATGT | GCTTTCAAGAAAAAACCTCGA |
| *cdr-1* | TCTTCTCTCAATTGGCAACTG | TTTGGGTAAACTTCATGACGA |
| *cdf-2* | ATTGCCACGATGAAGCTGAC | CCACCAATAACTTCGCAAACC |
| *ttm-1* | TGCATTTGGCACTTGAATCT | CAGTTTCCTGTTGTTGACACG |
| *act-1* | GCTGGACGTGATCTTACTGATTACC | GTAGCAGAGCTTCTCCTTGATGTC |
| *tba-1* | GTACACTCCACTGATCTCTGCTGACAAG | CTCTGTACAAGAGGCAAACAGCCATG |
| *ubc-2* | AGGGAGGTGTCTTCTTCCTCAC | CGGATTTGGATCACAGAGCAGC |
| 18S rRNA (human) | GCCGCTAGAGGTGAAATTCTTG | CTTTCGCTCTGGTCCGTCTT |
| GAPDH (human) | GGCCTCCAAGGAGTAAGACC | AGGGGAGATTCAGTGTGTG |
| GUSB (human) | GAAAATATGTGGTTGGAGAGCTCATT | CCGAGTGAAGATCCCCTTTTTA |
| MT1X (human) | ACCACGCTTTTCATCTGTCC | GAGCAGTTGGGGTCCATTTC |
| MT2A (human) | AACCTGTCCCGACTCTAG | GAAGTCGCGTTCTTTACA |
| 18s  rRNA (mouse) | AGTCCCTGCCCTTTGTACACA | CGATCCGAGGGCCTCACTA |
| Mt1 (mouse) | CCTTCTCCTCACTTACTCCGTAGC | GGAGCCGCCGGTGGA |
| Mt2 (mouse) | TCCTGTGCCTCCGATGGAT | TGCAGGAAGTACATTTGCATTGT |
| Slc30a8 (mouse) | TGCACAGTCTACACATCTGGTCACT | TGGCTGGCAGCTGTAGCA |
| Med15 (mouse) | TCTTCCAACCAAACAGCAGG | TGGTTGAAGACAGGTGAACG |
| ChIP | | |
| MT2A promoter (human) | GGTGGTCAAGAGGTGTTTACTT | GACTCTTGGATTGGTGTCTCTG |
| MT2A  (-10kb) (human) | TGCGGAACTGTGAGTCAATTA | AGAGGAGAGAGTGAGCAAGT |
| MT1X promoter (human) | CGAGGTGGAGCCAAAGG | GCAAGGAGAAGCAGGAGTT |
| MT1X  (-10kb) (human) | TTCTCTTCTCGCTTGGGAAC | AAGCAGCGGAGGAAGTAAAG |
| Mt1 promoter (mouse) | CGGACTCGTCCAACGACTATAA | CGCCAACTAAAGGTGCCTATTC |
| Mt1 (+10kb) (mouse) | CCAGAACAACCCAGTCCTAAA | AGGGTCTTGCTCTCCAGATA |
| Slc30a8 enhancer (mouse) | GAAACAGGGCAGGTACTCAA | TTACAGCGAGAGGCAAGTAAG |
| Slc30a8  (-10kb) (mouse) | AAGATGTGGCTCAGTGGTAAA | GCCTGTCTCATGGGTCTTATT |
